# HLF transactivates *TFEB* to promote gallbladder cancer stem cells’ self-renewal and determines tumor response to distinct therapies

**DOI:** 10.1126/sciadv.adv6723

**Published:** 2025-08-08

**Authors:** Daimin Xiang, Zhao Yang, Mingye Gu, Midie Xu, Chunliang Liu, Erdong Liu, Junyu Liu, Yichuang Wang, Hongyang Wang, Jing Fu

**Affiliations:** ^1^ International Cooperation Laboratory on Signal Transduction, National Center for Liver Cancer, Third Affiliated Hospital of Naval Medical University, Shanghai 200438, China.; ^2^ Medical Innovation Center, Shanghai East Hospital, School of Medicine, Tongji University, Shanghai 200120, China.; ^3^ Department of Hepatobiliary Surgery, Third Affiliated Hospital of Naval Military Medical University, Shanghai 200438, China.; ^4^ State Key Laboratory of Oncogenes and Related Genes, Shanghai Cancer Institute, Renji Hospital, Shanghai Jiao Tong University School of Medicine, Shanghai 200127, China.; ^5^ Department of Pathology, Fudan University Shanghai Cancer Center, Shanghai 200032, China.

## Abstract

Gallbladder cancer (GBC) is the most common malignancy in the biliary system and lacks biomarkers for personalized therapy. Here, we reported that hepatic leukemia factor (HLF) was highly expressed in gallbladder cancer stem cells (CSCs) and patients with gemcitabine-resistant GBC. Mechanistic study revealed that interleukin-6 receptor (IL-6R) and transcription factor EB (TFEB) are direct target genes of HLF. The IL-6/IL-6R/signal transducer and activator of transcription 3 axis transactivates HLF expression in GBC, forming a positive feedback loop. Functional studies revealed that HLF promoted gallbladder CSCs’ expansion and gemcitabine resistance via TFEB-induced autophagy. In addition, HLF drives TFEB-induced programmed death ligand 1 expression in human tumors and governs tumor immune evasion in a CD8^+^ T cell–dependent manner. Patient cohorts’ analysis suggested that HLF levels in GBCs might determine the distinct responses to chemotherapy and immunotherapy. In conclusion, our findings demonstrated that HLF could act as a driver for gallbladder CSCs’ self-renewal and drug resistance and a biomarker for individualized therapy.

## Teaser

HLF predicts GBC-personalized therapy.

## INTRODUCTION

Gallbladder cancer (GBC) is one of the most aggressive and fatal malignancies (*1*). Cholelithiasis is a cofactor in GBC development, and inflammatory factors play a vital role in this transformation process (*2*). Most patients with GBC have no obvious symptoms at the early stages of the disease and progress to the middle and late stages when diagnosed, thereby losing the opportunity for surgical resection. The 5-year survival rate of patients with stage IV GBC is less than 5%, even if radical resection is performed (*3*). Although conventional chemotherapy and immunotherapy have been used as first-line therapies for GBC, there has been no definitive improvement in overall survival (OS) (*4*, *5*). Therefore, it is urgent to explore the pathogenesis and drug resistance mechanism of GBC.

Autophagy is an evolutionarily conserved catabolic process that transports damaged or necrotic substances from the cytoplasm to lysosomes for catabolism. This process is initiated and tightly regulated by several autophagy-related genes (ATGs) (*6*). Accumulating evidence suggests that autophagy is not only involved in the occurrence and development of tumors but is also closely related to tumor immunity (*7*, *8*). Autophagy also plays multifaceted roles in GBC, including promoting tumor growth, enhancing metastasis, and mediating chemotherapeutic resistance (*9*, *10*). Inhibition of autophagy by regulating ATGs has proven to be an effective treatment regimen for tumor progression and drug resistance (*11*). However, dysregulation of ATGs and their upstream regulators in GBC remains poorly understood.

The interaction between programmed death ligand 1 (PD-L1; also called CD274 or B7-H1) on tumor cells and programmed cell death 1 (PD-1) on activated T cells regulates immune evasion, leading to T cell exhaustion (*12*). The PD-1/PD-L1 immune checkpoint blockade markedly augments T cell responses and displays certain clinical therapeutic effects in several advanced cancers (*13*, *14*). The latest phase 3 clinical studies showed that immunotherapy plus gemcitabine and cisplatin improved OS versus placebo plus gemcitabine and cisplatin in advanced biliary tract cancer (*4*, *5*). Approximately 30% of patients with advanced GBC benefit from anti–PD-(L)1 treatment, with a complete or partial response (*15*). Tumor PD-L1 expression is considered a potential efficacy biomarker; however, the complex mechanisms underlying its regulation have not been completely clarified. Therefore, it is critical to delineate the molecular mechanism of PD-L1 expression to select the agent(s) that are likely to exhibit therapeutic activity against individual patient tumors.

Hepatic leukemia factor (HLF), a member of the proline and acidic amino acid–rich basic leucine zipper family of transcription factors, was first identified in acute lymphoblastic leukemia in the form of e2A-HLF fusion gene (*16*). Wild-type HLF forms homodimers or heterodimers with other proline and acidic amino acid-rich (PAR) factors and specifically binds to DNA for the transcriptional regulation of downstream molecules (*16*, *17*). *HLF* has been reported to be dysregulated in human tumors, functioning as an oncogene or tumor suppressor depending on the context. Our previous study showed that HLF was highly expressed in activated stellate cells and promoted liver fibrosis (*18*). We also identified that *HLF* works as an oncogene in hepatocellular carcinoma, intrahepatic cholangiocarcinoma, and triple-negative breast cancer (*19*–*21*). However, the roles of HLF in GBC progression, chemoresistance, and immune evasion remain unclear.

In this study, we found that HLF drives gallbladder CSCs’ self-renewal, chemoresistance, and immune evasion by transactivating transcription factor EB (*TFEB*), suggesting that HLF is a promising therapeutic target and biomarker for GBC.

## RESULTS

### HLF is up-regulated in gallbladder CSCs and indicates poor prognosis of patients with GBC

**Fig. 1.**
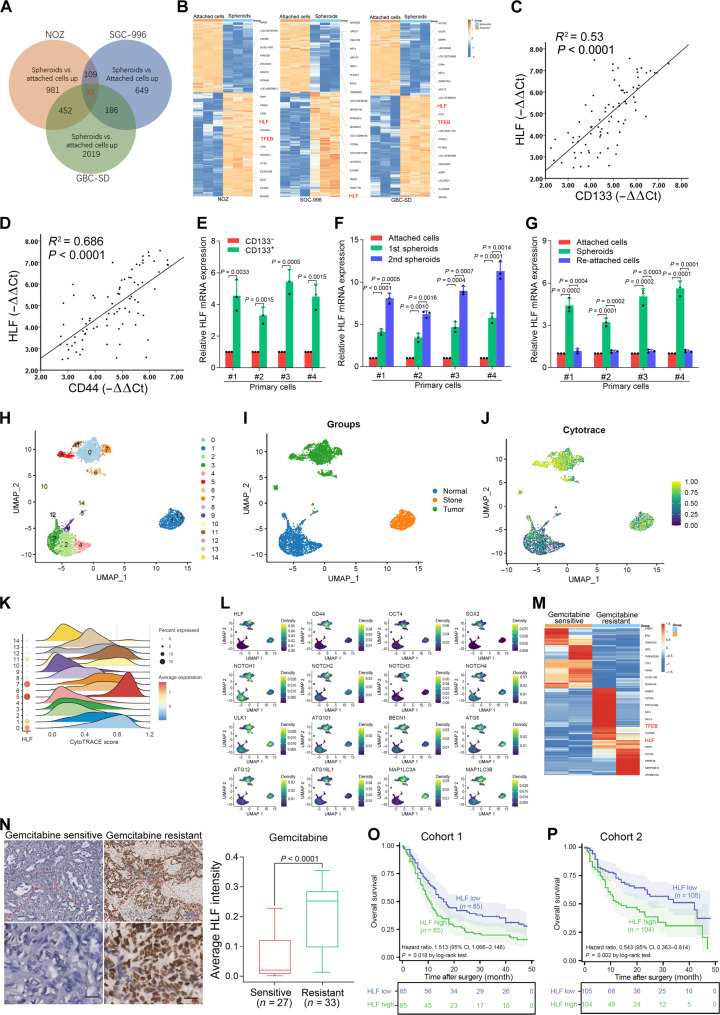
HLF is up-regulated in gallbladder CSCs and indicates poor prognosis of patients with GBC. (**A**) Venn diagrams showing the up-regulated genes in GBC spheroids compared to GBC attached cells. A total of 93 genes that are commonly up-regulated in both datasets. GBC-SD, NOZ, and SGC-996 are GBC cell lines. (**B**) Heatmap showing RNA differential expression of genes between GBC spheroids and GBC attached cells. NOZ (left), SGC-996 (middle), and GBC-SD (right). (**C** and **D**) The correlation between the level of HLF and CD133 or CD44 in GBC tissues (*n* = 80) was determined by real-time polymerase chain reaction (PCR) analysis. *R*^2^, coefficient of determination. (**E**) Real-time PCR analysis of HLF expression in sorted CD133^+^ primary GBC cells and negative cells (*n* = 3). (**F**) Real-time PCR analysis of HLF expression in serial passages of primary GBC spheroids (*n* = 3). (**G**) Real-time PCR analysis of HLF expression in primary GBC adherent, spheres, and readherent cells (*n* = 3). (**H** and **I**) Uniform manifold approximation and projection (UMAP) showing Seurat clusters and sample types of 4562 epithelial cells. (**J**) CytoTRACE analyzes the stemness of cells, with the color bar on the right illustrating the correspondence between colors and stemness scores. (**K**) HLF expression levels and stemness scores across individual clusters. The bubble plot (left) shows HLF expression. The ridgeline plot (right) displays the distribution of stemness scores. (**L**) The UMAP density visualization depicts expression patterns of HLF and canonical stemness-associated genes or autophagy-associated genes. (**M**) Heatmap showing RNA differential expression of genes between gemcitabine-sensitive or -resistant GBC PDX tissues. (**N**) Immunohistochemical (IHC) staining of HLF in gemcitabine-sensitive or -resistant GBC tissues. Scale bars, 5 μm (blue) and 25 μm (black). (**O** and **P**) IHC staining analysis of HLF was performed in patients with GBC (cohort 1, *n* = 170; cohort 2, *n* = 209). Kaplan-Meier analysis for OS was performed according to HLF levels. CI, confidence interval.

To screen for potential targets that may be associated with cancer stem cell (CSC) properties in GBC, we enriched the gallbladder CSCs by inducing GBC spheroid formation in low-adhesion culture and performed mRNA profile analysis. Transcriptomic analysis showed that expression levels of 93 genes were commonly increased (greater than twofold; Fig. 1A), among which HLF was one of the top up-regulated genes (Fig. 1B). It is well established that CD133 and CD44 are gallbladder CSC markers (*22*). Notably, HLF levels were positively associated with the expression of CD133 and CD44 in GBC tissues (Fig. 1, C and D). On this basis, we enriched gallbladder CSCs by flow cytometry sorting or sphere formation from primary GBC cells and GBC cell lines. As expected, expression of HLF was markedly increased in sorted CD133^+^ primary GBC cells (Fig. 1E). Consistently, HLF levels were much higher in self-renewing spheroids than in the attached cells (fig. S1A). The level of HLF expression gradually increased over serial passages of primary GBC spheroids (Fig. 1F). HLF levels could be partially restored during reattachment in parallel with differentiation (Fig. 1G). Moreover, GBC cell lines showed similar results (fig. S1, B to F).

Next, we analyzed gallbladder samples at different stages through single-cell sequencing, yielding 13,359 high-quality single cells after stringent quality control screening (fig. S1G). On the basis of the expression profiles of cell type–specific marker genes [e.g., CD3D for T lymphocytes, CD79A for B lymphocytes, marginal zone and B1 cell-specific protein (MZB1) for plasma cells, CD68 for myeloid cells, tryptase alpha/beta-1 precursor (TPSAB1) for mast cells, Collagen I (COL1A1) for mesenchymal cells, Platelet And Endothelial Cell Adhesion Molecule 1 (PECAM1) for endothelial cells, and epithelial cell adhesion molecule (EPCAM) for epithelial cells], we systematically identified eight major cell populations (fig. S1H). Cross-sample compositional visualization revealed notable interindividual cellular heterogeneity (fig. S1I). To investigate the biological relationship between HLF and tumor stemness, we further subclassified epithelial cells via unsupervised clustering, resulting in 15 transcriptionally distinct subclusters (Fig. 1H). Uniform manifold approximation and projection (UMAP) dimensionality reduction demonstrated spatially dispersed distributions of epithelial cells across samples (Fig. 1I), indicating substantial intersample heterogeneity. Subsequent quantification using the CytoTRACE stemness scoring system revealed elevated HLF expression in subclusters with higher stemness scores (Fig. 1, J and K). Notably, classical stemness markers and autophagy-associated genes [e.g., transcription factor SOX-2 (SOX2), octamer-binding transcription factor 4 (OCT4), Beclin1 (BECN1), and ATG5] exhibited notable coexpression with HLF in identical subclusters (Fig. 1L), suggesting that HLF may participate in regulating the molecular mechanisms underlying epithelial cell stemness maintenance. We performed gene set variation analysis (GSVA) using the HALLMARK gene sets from MSigDB to investigate downstream regulatory pathways in three HLF-high subclusters (clusters 0, 5, and 7). Cluster 7 exhibited enrichment of apoptotic pathways, aligning with our experimental observations of enhanced apoptosis in this population. All three subclusters showed activation of stemness-associated pathways including Hedgehog signaling, Notch signaling, WNT/β-catenin signaling, and MYC targets (fig. S1J).

Existing evidence showed that CSCs are closely associated with tumor recurrence, metastasis, and chemoresistance (*23*). As expected, HLF expression was markedly up-regulated in recurrent GBCs or metastatic tissues compared with the primary lesions (fig. S1, K and L). Moreover, mRNA profile analysis showed that HLF expression was much higher in gemcitabine-resistant GBC patient-derived xenograft (PDX) tissues than gemcitabine-sensitive GBC PDX tissues (Fig. 1M). Consistently, HLF expression was much higher in tissues from a patient with gemcitabine-resistant GBC than tissues from a patient with gemcitabine-sensitive GBC (Fig. 1N). Two independent cohorts of patients with GBC (cohort 1, *n* = 170; cohort 2, *n* = 209) were recruited to investigate the clinical significance of HLF in GBC. HLF overexpression was positively associated with gallbladder stone, histological differentiation, lymph node metastasis, and tumor-nodes-metastasis stage (tables S1 and S2), suggesting that it plays a vital role in GBC progression. The Kaplan-Meier analysis revealed a correlation between high HLF levels and poor OS (Fig. 1, O and P).

### IL-6/IL-6R/phosphorylated signal transducer and activator of transcription 3 axis activates HLF in GBC cells

**Fig. 2.**
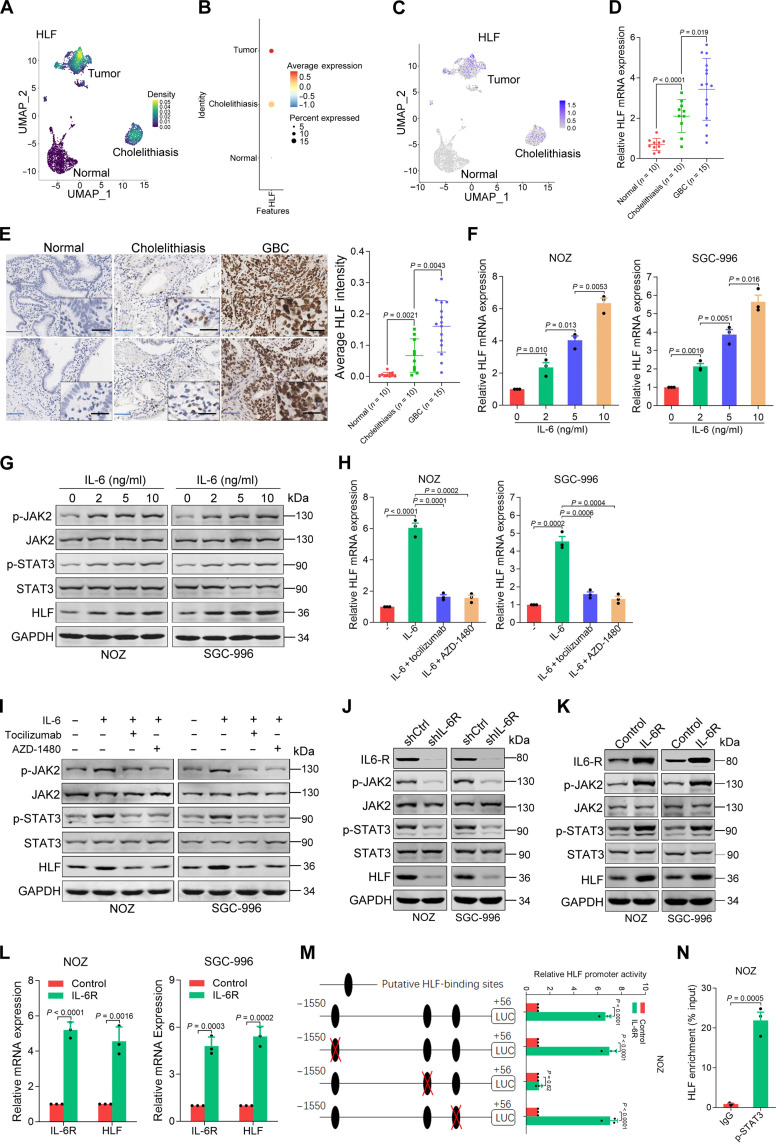
IL-6/IL-6R/STAT3 axis transactivates HLF expression in GBC cells. (**A**) Density map of three groups (normal, cholelithiasis, and GBC tissues). (**B**) Bubble plots of HLF expressed in the three groups (normal, cholelithiasis, and GBC tissues). (**C**) The expression of HLF on the UMAP plot (normal, cholelithiasis, and GBC tissues). (**D** and **E**) Real-time PCR analysis and IHC staining of HLF expression in adjacent nontumorous (*n* = 10), cholelithiasis (*n* = 10), and GBC tissues (*n* = 15). Scale bars, 5 μm (blue) and 25 μm (black). (**F**) NOZ/SGC-996 cells starved overnight were treated with IL-6 for 48 hours, followed by real-time PCR analysis (*n* = 3). (**G**) NOZ/SGC-996 cells starved overnight were treated with IL-6 for 48 hours, followed by Western blot analysis. GAPDH, glyceraldehyde-3-phosphate dehydrogenase. (**H** and **I**) NOZ/SGC-996 cells starved overnight were pretreated with AZD1480 (2 μM) or tocilizumab (20 μg/ml) for half an hour and were then stimulated with IL-6 (10 ng/ml) for an additional 48 hours, followed by real-time PCR analysis (*n* = 3) and Western blot analysis. Tocilizumab is an IL-6R inhibitor. AZD1480 is a JAK2/STAT3 inhibitor. (**J**) Western blot analysis of the protein expression of HLF, phosphorylated JAK2 (p-JAK2), and phosphorylated STAT3 (p-STAT3) in short hairpin IL-6R (shIL-6R) or short hairpin control (shCtrl) GBC cells. (**K**) Western blot analysis of the protein expression of HLF, p-JAK2, and p-STAT3 in IL-6R overexpression or control GBC cells. (**L**) Real-time PCR analysis of the mRNA expression of HLF and IL-6R in IL-6R overexpression or control GBC cells (*n* = 3). (**M**) Selective mutation analysis identified STAT3-responsive regions in the *HLF* promoter. Serially mutated *HLF* promoter constructs were transfected into NOZ IL-6R–overexpressing and control cells, and relative luciferase (LUC) activities were determined (*n* = 3). (**N**) NOZ cells were subjected to ChIP assay with anti-STAT3(Y705) or anti–immunoglobulin G (IgG) antibody, followed by real-time PCR (*n* = 3).

Notably, our single-cell transcriptome analysis showed that HLF expression level was progressively increased in chronic cholelithiasis and GBC tissues compared with adjacent nontumorous tissues (Fig. 2, A to C, and fig. S2A). Real-time polymerase chain reaction (PCR) and immunohistochemical (IHC) staining further confirmed that the expression of HLF was up-regulated in chronic cholelithiasis and GBC tissues than in adjacent nontumorous tissues (Fig. 2, D and E). IL-6/signal transducer and activator of transcription 3 (STAT3) signaling pathway plays a vital role in inflammatory cancer transformation (*24*, *25*). Therefore, we examined the effects of inflammatory factor IL-6 on HLF expression in GBC cells. As expected, HLF expression was enhanced upon IL-6 treatment in GBC cells (Fig. 2, F and G). The IL-6–triggered HLF induction was attenuated by the selective IL-6R inhibitor tocilizumab or the Janus kinase 2 (JAK2)/STAT3 inhibitor AZD1480 (Fig. 2, H and I). Moreover, HLF expression in GBC cells decreased because of the knockdown of IL-6R (Fig. 2J and fig. S2B). Consistently, HLF expression was up-regulated by ectopic IL-6R expression in GBC cells (Fig. 2, K and L). Sequence analysis revealed three putative STAT3-binding sites in the HLF promoter (fig. S2C). Serial site–directed mutagenesis and deletions revealed that the second STAT3-binding site was critical for STAT3-induced HLF transactivation (Fig. 2M and fig. S2D). Significant enrichment of STAT3 in the HLF promoter region was detected by chromatin immunoprecipitation (ChIP)–quantitative PCR (qPCR; Fig. 2N and fig. S2E).

### HLF activates IL-6R and TFEB through transcription

**Fig. 3.**
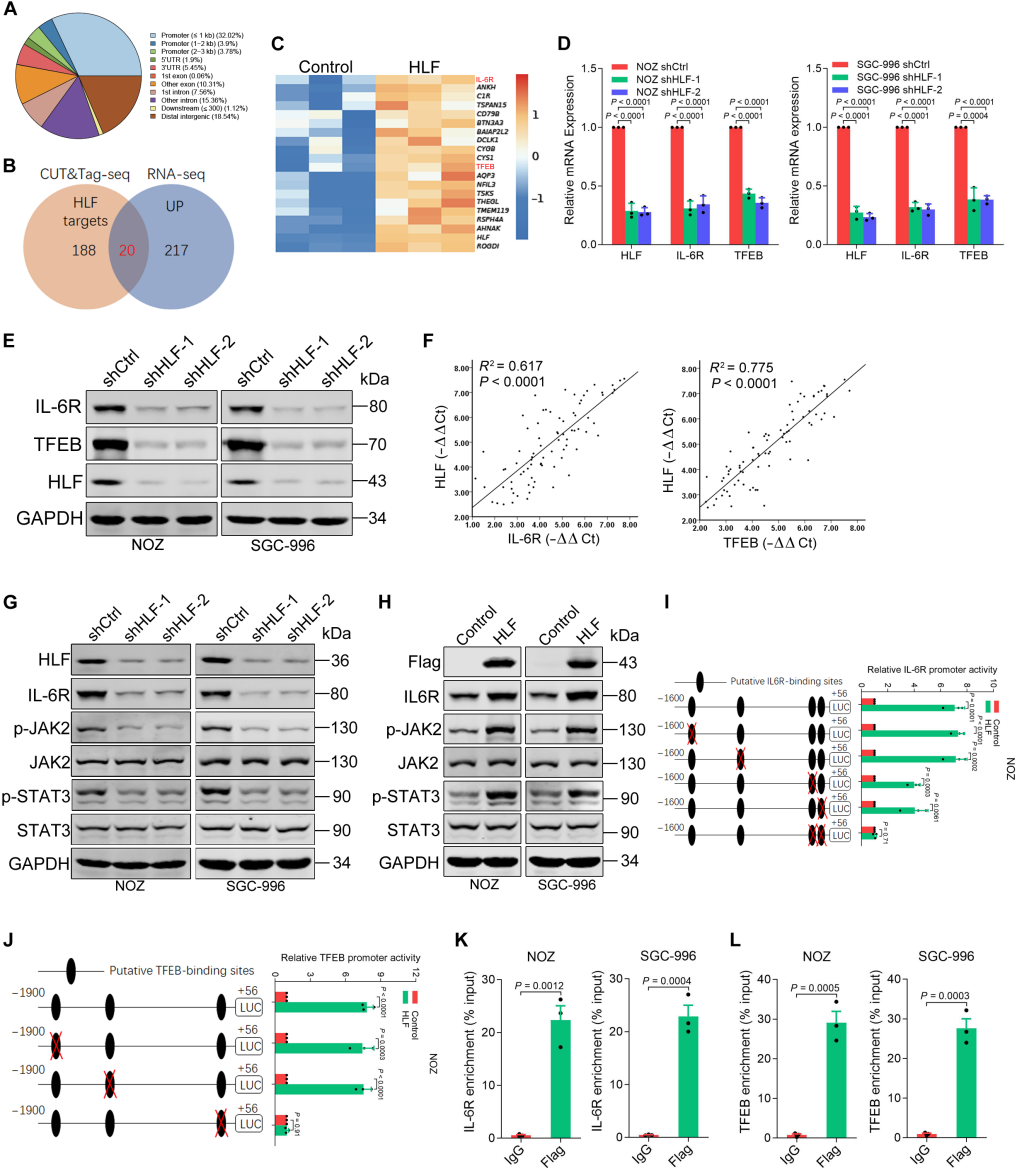
HLF transcriptionally activates IL-6R and TFEB in GBC cells. (**A**) CUT&Tag-seq dataset for HLF in NOZ cells was generated using the anti-Flag antibody. Pie chart represents the genome-wide distribution of HLF occupancy in promoter, exon, intron, and intergenic regions in NOZ cells, after peak calling. 5′UTR, 5′ untranslated region. (**B**) Venn diagram representing the overlap between HLF targets, identified by CUT&Tag, and RNA-seq (|fold change| >2 and *P* < 0.05). (**C**) Heatmap showing RNA-seq of up-regulated genes between NOZ HLF and NOZ control cells. (**D** and **E**) Real-time PCR and Western blot analysis of the expression of HLF, IL-6R, and TFEB in short hairpin HLF (shHLF) or shCtrl GBC cells. (**F**) The correlation between the level of HLF and IL-6R or TFEB in GBC tissues (*n* = 80) was determined by real-time PCR analysis. (**G**) Western blot analysis of the protein expression of IL-6R, p-JAK2, and p-STAT3 in shHLF or shCtrl GBC cells. (**H**) Western blot analysis of the protein expression of IL-6R, p-JAK2, and p-STAT3 in HLF-overexpressing or control GBC cells. (**I** and **J**) Selective mutation analysis identified HLF-responsive regions in the *IL-6R* or *TFEB* promoter. Serially mutated *IL-6R* or *TFEB* promoter constructs were transfected into NOZ HLF–overexpressing and control cells, and relative luciferase activities were determined (*n* = 3). (**K** and **L**) GBC cells were subjected to ChIP assay with anti-Flag or anti-IgG antibody, followed by real-time PCR (*n* = 3).

Next, we examined the HLF-binding sites from our Cleavage Under Targets & Tagmentation sequencing (CUT&Tag-seq) analysis, together with the HLF overexpression profiles from our RNA sequencing (RNA-seq) analysis (fold change > 1.5 and *P* < 0.05) (Fig. 3, A to C). Gene expression analysis using real-time PCR showed that the expression levels of interleukin-6 receptor (IL-6R) and TFEB were significantly altered (Fig. 3D and fig. S3A). Western blot analysis also confirmed that IL-6R and TFEB activation were impaired in HLF-knockdown GBC cells, while the opposite effect was observed when HLF was overexpressed (Fig. 3E and fig. S3B). Immunofluorescence staining further confirmed the consistent down-regulation of IL-6R and TFEB in HLF-knockdown GBC cells (fig. S3C). A decreased level of IL-6R and TFEB expression was detected in HLF-knockdown xenografts (fig. S3D). Notably, a correlation between HLF expression and the levels of IL-6R or TFEB was observed using real-time PCR in GBCs from 80 patients (Fig. 3F). The IL-6/IL-6R complex initiates dimerization of Glycoprotein 130 (gp130), which triggers its activation downstream of JAK and STAT3 (*26*). As expected, phosphorylation of JAK and STAT3 was impaired in HLF-knockdown GBC cells (Fig. 3G) and enhanced in HLF-overexpressing GBC cells (Fig. 3H). Furthermore, sequence analysis revealed four and three putative HLF-binding sites in the IL-6R and TFEB promoters, respectively (fig. S3E). Serial site–directed mutagenesis and deletions showed that the third and fourth HLF-binding sites were critical for HLF-induced IL-6R transactivation and that the third HLF-binding site was critical for HLF-induced TFEB transactivation (Fig. 3, I and J, and fig. S3, F and G). Significant enrichment of HLF in the promoter region of either IL-6R or TFEB was detected by ChIP-qPCR (Fig. 3, K and L). Together, these results suggest that HLF transactivates IL-6R and TFEB expression in GBC cells.

### HLF promotes gallbladder CSCs’ self-renewal

Next, we observed that GBC cells with HLF knockdown exhibited reduced expression of CSC markers and pluripotent transcription factors and impaired spheroid formation (fig. S4, A to C). Consistently, enforced HLF expression increased amounts of CSC markers and pluripotent transcription factors and enhanced spheroid forming ability of GBC spheres (fig. S4, D to F). Moreover, in vitro limiting dilution assays showed that the proportion of gallbladder CSCs was reduced in HLF-knockdown GBC cells and increased in HLF-overexpressing GBC cells (fig. S4, G and H). In vivo limiting dilution assays revealed that interference with HLF markedly reduced tumor incidence (fig. S4, I and J). Consistently, HLF overexpression markedly enhanced tumor incidence (fig. S4, K and L).

### TFEB exerts oncogenic effects and promotes autophagy in GBC cells

TFEB is a central regulator of the autophagic process (*27*). Therefore, we examined the effects of TFEB on autophagy in GBC cells. TFEB depletion decreased light chain 3 (LC3) II and enhanced the levels of p62 in GBC cells (fig. S5A). In contrast, TFEB overexpression increased the levels of LC3 II and reduced p62 expression in GBC cells (fig. S5B). Transmission electron microscopy (TEM) revealed that autophagosomes were reduced in TFEB-depleted GBC cells (fig. S5C) and increased in TFEB-overexpressing GBC cells (fig. S5D). Furthermore, the rate of autophagic flux, as illustrated by live-cell imaging using an monomeric red fluorescent protein–green fluorescent protein–microtubule-associated protein 1 light chain 3 (mRFP-GFP-LC3) reporter construct, was inhibited by TFEB knockdown and enhanced by TFEB overexpression (fig. S5, E to H).

Next, we investigated the effect of TFEB on gallbladder CSCs’ self-renewal and chemoresistance. The results showed that TFEB depletion inhibited the self-renewal and tumorigenicity of GBC cells (fig. S6, A to C). Consistently, GBC cells’ self-renewal and tumorigenicity were promoted by overexpression of TFEB (fig. S6, D to F). On the basis of these results, we concluded that TFEB is an oncogene and a positive modulator of autophagy in GBC.

### HLF promotes gallbladder CSCs’ self-renewal via TFEB-mediated autophagy

**Fig. 4.**
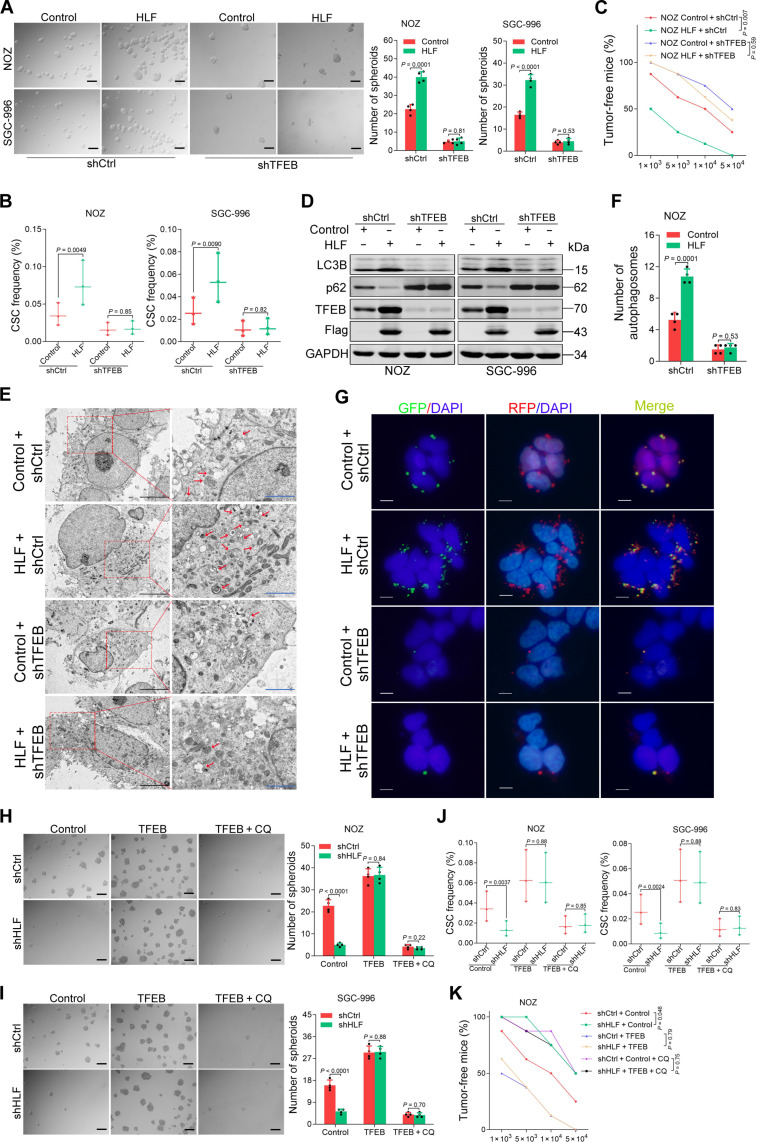
HLF regulates gallbladder CSCs’ self-renewal via TFEB-induced autophagy. (**A** to **C**) HLF-overexpressing and control cells infected with *TFEB* knockdown or control virus were subjected to spheroid formation (A), in vitro limiting dilution assays (B), and in vivo limiting dilution assays (C). (**D**) NOZ/SGC-996 HLF and control cells infected with TFEB knockdown or control virus were subjected to Western blot assay. LC3B and p62 are autophagy markers. (**E** and **F**) NOZ HLF and control cells infected with TFEB knockdown or control virus. Then, the autophagosomes were observed under the TEM. Red arrows indicate the autophagosomes. Scale bars, 2 μm (blue) and 5 μm (black). (**G**) The effect of HLF on promoting autophagy was blocked by short hairpin TFEB (shTFEB), as assessed by confocal imaging in NOZ cells expressing mRFP-GFP-LC3. Scale bars, 5 μm. (**H** to **J**) shHLF and control cells were infected with *TFEB* overexpression virus. The indicated cells were subjected to spheroid formation (H and I) and in vitro limiting dilution assays (J) in the presence and absence of CQ (10 μM) for 7 days. CQ is an autophagy inhibitor. (**K**) NOZ shHLF and control cells were infected with *TFEB* overexpression virus. The indicated cells were subjected to in vivo limiting dilution assays in the presence and absence of CQ. CQ (25 mg/kg) was injected intraperitoneally once daily for 1 week.

We further explored whether HLF promotes gallbladder CSCs’ self-renewal via TFEB. As expected, the knockdown of TFEB diminished the difference in the degree of self-renewal and tumorigenicity between HLF-overexpressing and control GBC cells (Fig. 4, A to C, and fig. S7A). The effect of HLF overexpression on the promotion of autophagy was reversed by TFEB knockdown (Fig. 4, D to G, and fig. S7, B to G). Consistently, autophagy inhibition mediated by HLF depletion was restored by TFEB overexpression (fig. S7, H to L). Furthermore, the self-renewal and tumorigenicity of HLF-knockdown GBC cells were restored through the introduction of TFEB (Fig. 4, H to K, and fig. S7, M to P), while autophagy inhibitor chloroquine (CQ) or bafilomycin A1 could abolish this restored effect (Fig. 4, H to K, and fig. S7, M to P). Overall, TFEB-mediated autophagy partially contributes to the functional effects of HLF on GBC cells.

### HLF/TFEB/autophagy axis determines the response of gemcitabine in GBC

**Fig. 5.**
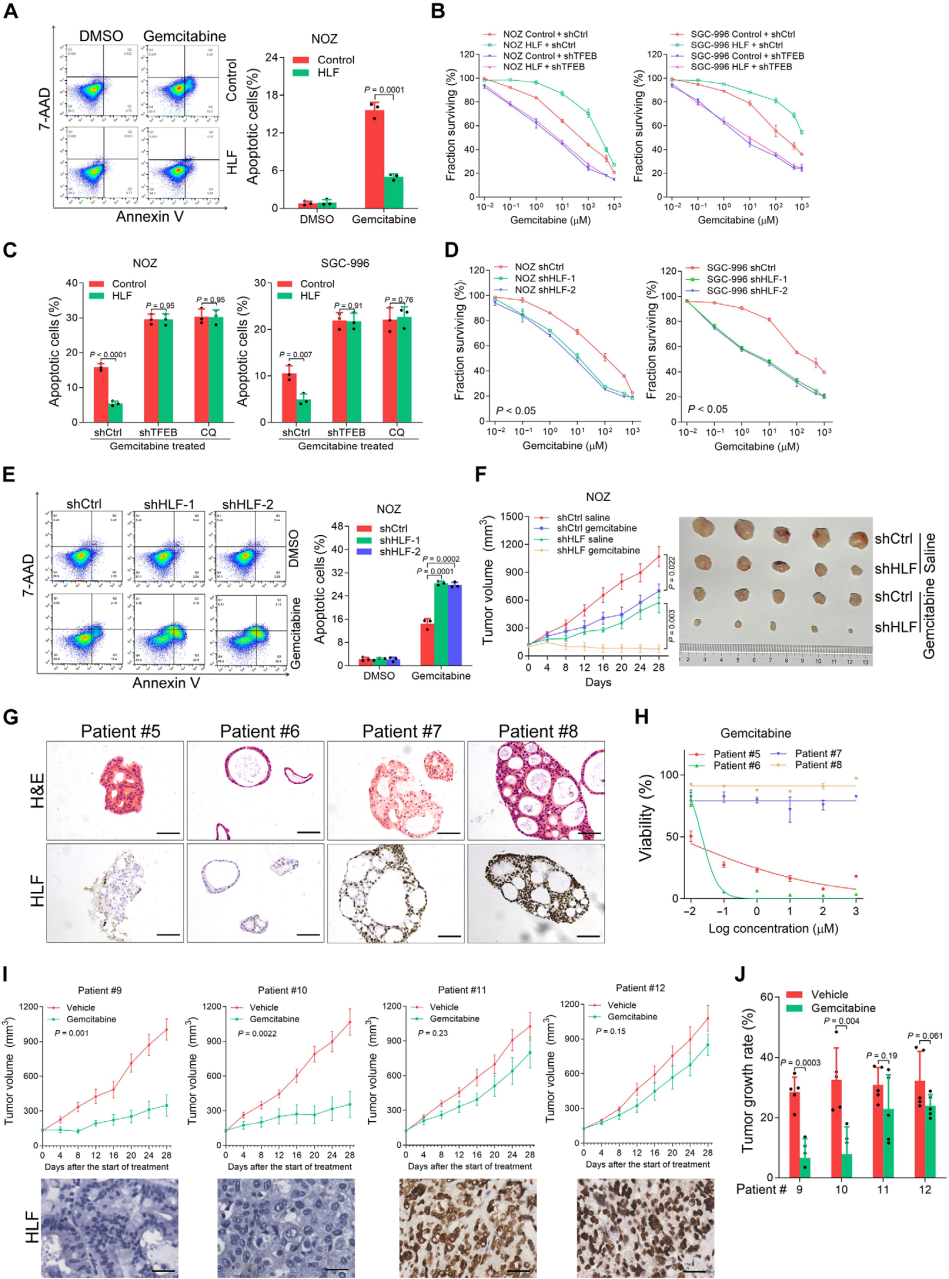
HLF/TFEB/autophagy axis determines the response of gemcitabine in GBC. (**A**) HLF-overexpressing and control NOZ cells were treated with gemcitabine for 48 hours, and apoptosis was examined by flow cytometry (*n* = 3). (**B**) HLF-overexpressing and control cells were infected with *TFEB* knockdown or control virus. The cells were then treated with gemcitabine (0, 0.01, 0.1, 1, 10, 100, 500, and 1000 μM) for 48 hours, and cell survival curves were calculated. (**C**) HLF-overexpressing and control cells were infected with *TFEB* knockdown virus or treated with CQ. The cells were then treated with gemcitabine for 48 hours, and their apoptosis was examined by flow cytometry (*n* = 3). (**D**) HLF-knockdown and control GBC cells were treated with gemcitabine (0, 0.01, 0.1, 1, 10, 100, 500, and 1000 μM) for 48 hours, and their cell survival curves were calculated. (**E**) HLF-knockdown and control GBC cells were treated with gemcitabine for 48 hours, and apoptosis was examined by flow cytometry (*n* = 3). 7-AAD, 7-Aminoactinomycin D. (**F**) Tumor growth curves of mice after the subcutaneous injection of the NOZ shHLF or control cells and intraperitoneal administration of gemcitabine (*n* = 5). (**G** and **H**) PDOs derived from the primary GBCs with low HLF levels (patients #5 and #6) or high HLF levels (patients #7 and #8) were treated with gemcitabine for 10 days, and their cell survival curves were calculated. Scale bars, 100 μm. H&E, hematoxylin and eosin. (**I** and **J**) PDXs derived from the primary GBCs with low HLF levels (patients #9 and #10) or high HLF levels (patient #11 and #12) were treated with gemcitabine or saline for 4 weeks (*n* = 5). The xenograft growth was monitored. Scale bars, 25 μm. The tumor growth rate of PDXs was quantified.

Considering that CSCs have been found to be closely associated with the resistance of cancer to pharmacotherapy, we next investigated the correlation between HLF expression and gemcitabine response in GBC cells. As expected, HLF-overexpressing GBC cells were more resistant to gemcitabine than control cells, as shown by an increase in median inhibitory concentration (fig. S8A). HLF overexpression markedly decreased the gemcitabine-induced GBC cell apoptosis (Fig. 5A and fig. S8B). TFEB knockdown reduced gemcitabine resistance in HLF-overexpressing GBC cells (Fig. 5, B and C). Moreover, the interference of HLF sensitized GBC cells to gemcitabine-induced growth inhibition and cell apoptosis (Fig. 5, D and E, and fig. S8C). Consistent with the in vitro findings, intraperitoneal gemcitabine administration showed an improved therapeutic effect on the GBC xenografts with HLF interference (Fig. 5F).

Next, patient-derived tumor organoids (PDOs) and PDXs were established using fresh patient GBC tissues to validate the correlation between HLF levels and gemcitabine response. We found that PDOs or PDXs derived from tumors with low HLF levels were sensitive to gemcitabine treatment (Fig. 5, G to J). Moreover, the decreased expression of Ki-67, a marker of proliferating cells, was only detected in PDXs administered gemcitabine if they were derived from tumors with low HLF levels (fig. S8, D and E). Together, these results demonstrate that the HLF/TFEB/autophagy axis determines the response of GBC to gemcitabine.

### HLF/TFEB axis up-regulates PD-L1 and suppresses anticancer immunity

**Fig. 6.**
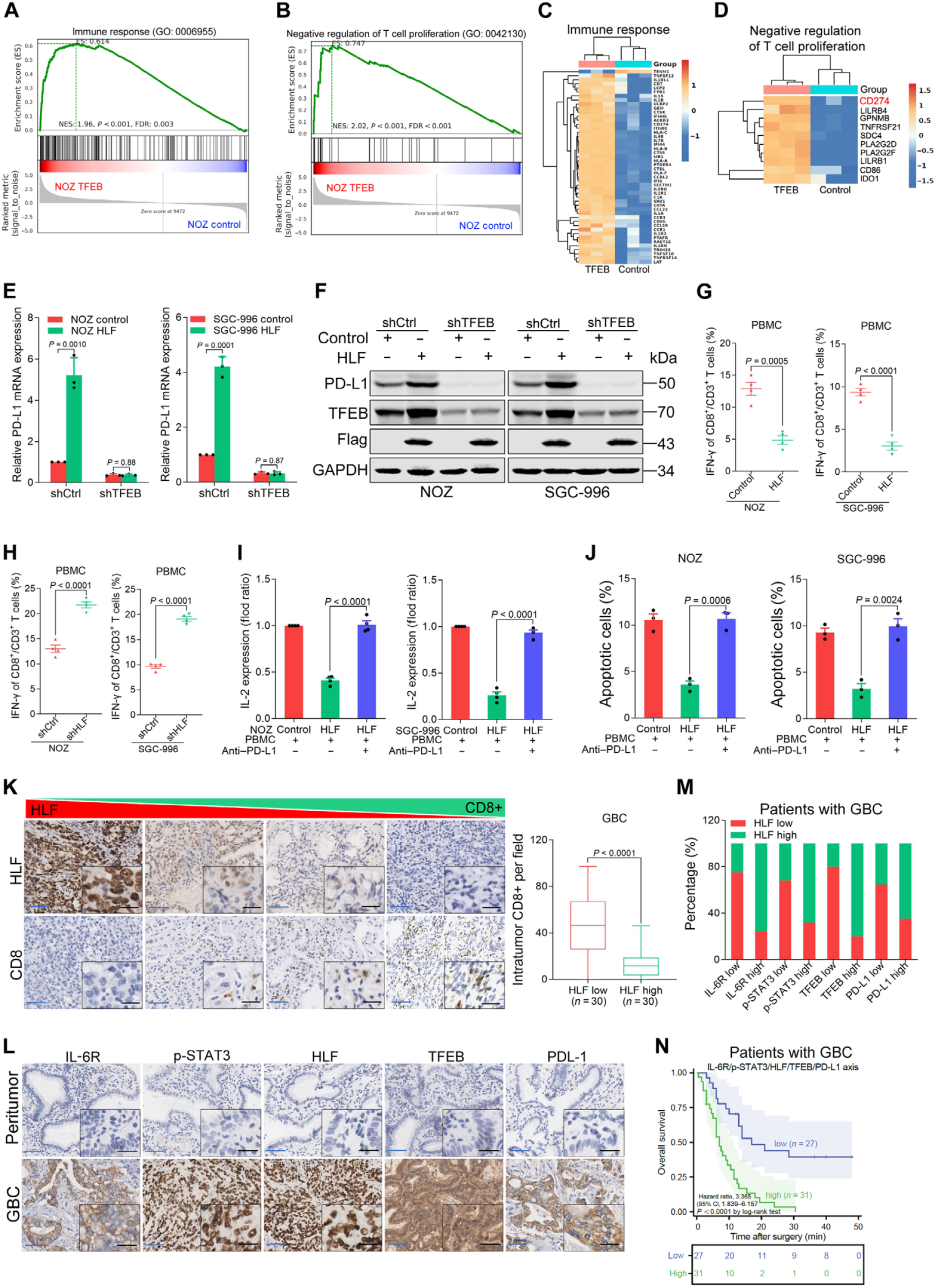
HLF activation promotes immune evasion of tumor cells. (**A** and **B**) GO analysis shows the enrichment of gene sets positively related to glycolysis, immune response or negative regulation of T cell proliferation in NOZ TFEB compared with control cells. FDR, false discovery rate. NES, normalized enrichment score. (**C** and **D**) Heatmap showing RNA differential gene expression of immune response or negative regulation of T cell proliferation between NOZ TFEB and control cells. (**E** and **F**) NOZ/SGC-996 HLF and control cells infected with *TFEB* knockdown or control virus were subjected to real-time PCR and Western blot assay. (**G** and **H**) Activated peripheral blood mononuclear cells (PBMCs) were cocultured with the indicated GBC cells for 3 days at a 4:1 ratio of PBMCs to tumor cells. Quantification of intracellular interferon-γ (IFN-γ) in CD8^+^/CD3^+^ T cells (*n* = 4). (**I**) NOZ/SGC-996 HLF or control cells were cocultured with activated PBMCs and then treated for 3 days with the anti–PD-L1 antibody atezolizumab. Soluble IL-2 levels in the supernatant of the coculture system were assayed using ELISA (*n* = 3). (**J**) Apoptosis of GBC cells in the coculture system was assayed on the basis of staining with fluorescein isothiocyanate (FITC)–annexin V and propidium iodide (PI; *n* = 3). (**K**) Immunohistochemistry of clinical GBC samples reveals an inverse correlation between tumor HLF expression (top) and CD8^+^ T cell infiltration (bottom). Scale bars, 5 μm (blue) and 25 μm (black). (**L**) IHC staining of IL-6R, p-STAT3, HLF, TFEB, and PD-L1 expression in tumor and the corresponding peritumor tissues from patients with GBC (*n* = 170). Scale bars, 5 μm (blue) and 25 μm (black). (**M**) Correlation analysis of HLF expression with IL-6R, p-STAT3, TFEB, and PD-L1 expression in GBC tissues (*n* = 170). (**N**) Kaplan-Meier analysis of OS in patients with GBC (*n* = 58) according to the expression of the IL-6R/p-STAT3/HLF/TFEB/PD-L1 axis.

To investigate which genes may be differentially regulated in the presence of TFEB, we profiled gene expression in TFEB-overexpressing or control GBC cells using RNA-seq. Gene Ontology (GO) revealed that TFEB overexpression resulted in the enrichment of gene sets related to the immune response (Fig. 6, A to D). In particular, PD-L1, which plays a crucial role in the immune response, was among the up-regulated genes (Fig. 6, C and D). The up-regulated PD-L1 expression was further confirmed by real-time PCR and Western blotting (fig. S9, A and B). Sequence analysis revealed three putative TFEB-binding sites in the PD-L1 promoter (fig. S9C). Serial site–directed mutagenesis and deletions revealed that the second TFEB-binding site was critical for TFEB-induced PD-L1 transactivation (fig. S9, D and E). Significant enrichment of TFEB in the promoter region of PD-L1 was detected using ChIP-qPCR (fig. S9F). Moreover, we found that forced overexpression of HLF in GBC cells enhanced PD-L1 expression (Fig. 6, E and F). In contrast, PD-L1 expression was reduced in HLF-depleted GBC cells (fig. S9, G and H). Notably, HLF-triggered PD-L1 induction was attenuated by TFEB knockdown (Fig. 6, E and F). HLF depletion–mediated PD-L1 down-regulation was reversed by TFEB overexpression (fig. S9, G and H), suggesting that the HLF/TFEB axis activates PD-L1 in GBC.

To test whether HLF regulates antitumor T cell immunity by regulating PD-L1, we performed a T cell–mediated killing assay in vitro using a coculture system in which activated peripheral blood mononuclear cells (PBMCs) from healthy donors were cocultured with human GBC cell lines. GBC cells with HLF overexpression were more resistant to T cell death (fig. S9, I and J). In addition, the cytotoxic T cell activity of PBMCs was reduced in cocultures of GBC cells overexpressing HLF (Fig. 6G and fig. S9, K to M) and enhanced in cocultures of GBC cells with HLF depletion (Fig. 6H). Furthermore, treating PBMC/GBC cocultures with the anti–PD-L1 antibody atezolizumab markedly enhanced the release of IL-2 into the supernatant and promoted T cell–mediated killing (Fig. 6, I and J). It was observed that HLF expression inversely correlated with CD8^+^ T cell infiltration in human tumor clinical samples (Fig. 6K). These results suggest that the HLF/TFEB axis activates the PD-1/PD-L1 axis, which suppresses T cell function and facilitates GBC cells in escaping antitumor immune responses.

### IL-6R/p-STAT3/HLF/TFEB/PD-L1 axis is activated in GBC and predicts poor prognosis

Next, we investigated the protein expression levels of the IL-6R/phosphorylated STAT3 (p-STAT3)/HLF/TFEB/PD-L1 axis in tissues from patients with cancer. Similar to HLF, the protein levels of IL-6R, p-STAT3, TFEB, and PD-L1 were elevated in GBC tumors compared with the corresponding peritumor tissues (Fig. 6L and fig. S10A). Kaplan-Meier analysis revealed a correlation between high IL-6R, p-STAT3, TFEB, and PD-L1 levels and poor OS in patients with GBC (fig. S10, B to E). We further tested the correlation between these proteins in patients with GBC and found a positive correlation between HLF and IL-6R, p-STAT3, TFEB, and PD-L1 expression (Fig. 6M). Patients with high expression of the IL-6R/p-STAT3/HLF/TFEB/PD-L1 axis had significantly lower survival rates (Fig. 6N). Therefore, our patient data demonstrate that the IL-6R/p-STAT3/HLF/TFEB/PD-L1 axis is activated in GBC and correlates with a worse clinical outcome.

### HLF expression is associated with the efficacy of PD-(L)1 checkpoint blockade in human tumors

**Fig. 7.**
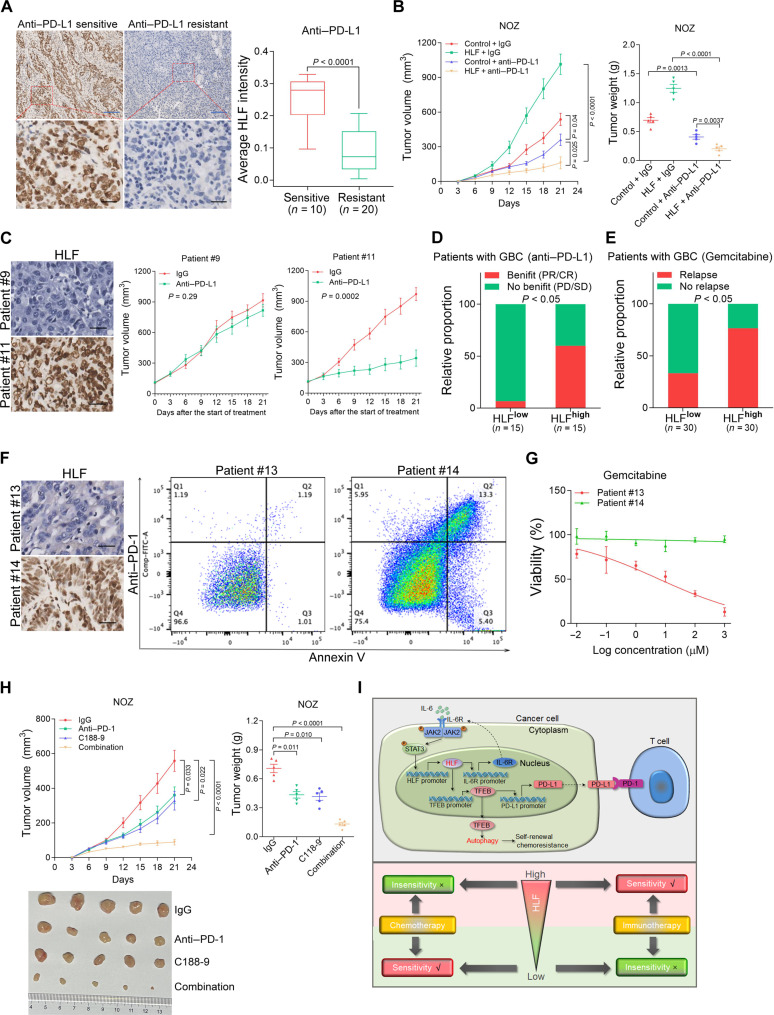
HLF expression is associated with the efficacy of PD-(L)1 checkpoint blockade in human tumors. (**A**) Representative images of IHC staining of HLF in anti–PD-L1–sensitive or anti–PD-L1–resistant GBC tissues. Scale bars, 5 μm (blue) and 25 μm (black). (**B**) HSC-NCG mice were injected subcutaneously with NOZ HLF and control cells with IgG control or anti–PD-L1 antibody treatment. Tumor volume and tumor weight are shown (*n* = 5). (**C**) HSC-NCG mice were inoculated with PDXs derived from the primary GBCs with low HLF levels (patient #9) or high HLF levels (patient #11). Then, the PDXs were treated with IgG control (IgG) or anti–PD-L1 antibody treatment for 3 weeks (*n* = 5). The xenograft growth was monitored. Scale bars, 25 μm. (**D**) Correlation analysis of HLF expression and responses of patients with GBC to anti–PD-L1 treatment (*n* = 30). (**E**) Correlation analysis of HLF expression and responses of patients with GBC to adjuvant gemcitabine treatment (*n* = 60). (**F**) PDOs derived from the primary GBCs with low HLF levels (patient #13) or high HLF levels (patient #14) were cocultured with activated PBMCs. Then, the PDOs were treated with IgG control or anti–PD-1 antibody treatment for 1 week. The PDO apoptosis was examined by flow cytometry. Scale bars, 25 μm. Compensated Fluorescein Isothiocyanate Area Signal (Comp-FTIC-A). (**G**) PDOs derived from the primary GBCs with low HLF levels (patient #13) or high HLF levels (patient #14) were cocultured with activated PBMCs. Then, the PDOs were treated with gemcitabine for 10 days, and their cell survival curves were calculated. (**H**) HSC-NCG mice were injected subcutaneously with NOZ cells with STAT3 inhibitor C188-9 and/or anti–PD-1 antibody treatment. Tumor volume, tumor weight, and tumor pictures are shown (*n* = 5). (**I**) Schematic model of the mechanism underlying HLF-driven gallbladder CSCs’ self-renewal, chemoresistance, and immune evasion.

Notably, we observed that HLF expression was much lower in tissues from a patient with anti–PD-L1–resistant GBC than tissues from a patient with anti–PD-L1–sensitive GBC (Fig. 7A). As expected, anti–PD-L1 administration enhanced the therapeutic effect in HLF-overexpressing GBC xenografts (Fig. 7B). The number of Ki-67–positive cells was much lower and CD8-positive or terminal deoxynucleotidyl transferase–mediated deoxyuridine triphosphate nick end labeling (TUNEL)–positive cells was much higher in the anti–PD-L1–treated HLF-overexpressing group than in the other groups (fig. S11A). In addition, there was no significant weight change among the different groups of mice during treatment (fig. S11B). Furthermore, CD34^+^ humanized NOD-Prkdce^m26Cd52^IL2rg^em26Cd22^/Gpt (NCG) mice with an intact immune system were inoculated with GBC PDXs. We found that PDXs derived from GBC tumors with high HLF levels were sensitive to anti–PD-L1 treatment (Fig. 7C and fig. S11C).

Next, we evaluated 30 patients with GBC pre- and post–anti–PD-L1 therapy. Nine of the 15 patients with GBC and high HLF expression benefitted from anti–PD-L1 treatment with a complete response (CR) or partial response (PR), whereas only one of 15 patients with GBC and low HLF expression responded to immune checkpoint blockade, and most had progressive disease (PD) (Fig. 7D). Consistently, the clinical cohort analysis showed that low TFEB expression levels were correlated with poor outcome (fig. S11D). Moreover, we evaluated 60 patients with GBC following postoperative adjuvant gemcitabine treatment. Twenty of the 30 patients with GBC and low HLF expression benefitted from adjuvant gemcitabine treatment, whereas seven of 30 patients with GBC and high HLF expression responded to gemcitabine, and most had relapse (Fig. 7E). Furthermore, PDOs were cocultured with activated PBMCs from corresponding patients with GBC. The results showed that PDOs derived from GBC tumors with high HLF levels were sensitive to anti–PD-1 treatment while resistant to gemcitabine treatment (Fig. 7, F and G, and fig. S11E). Together, these results suggested that GBC with low HLF-expressing levels was more sensitive to chemotherapy, while GBC with high HLF-expressing levels was more sensitive to immunotherapy.

As p-STAT3 is responsible for HLF/TFEB/PD-L1 activation, C188-9 was investigated in vivo. The combination of C188-9 and anti–PD-1 resulted in a more marked suppression of xenograft growth than C188-9 or anti–PD-1 alone (Fig. 7H). Immunohistochemistry revealed that tumor treatment with a combination of both inhibitors led to fewer Ki-67–positive cells and more CD8-positive or TUNEL-positive cells than treatment with a single inhibitor (fig. S11F). None of the treatments adversely affected weight, liver, or kidney function in tumor-bearing mice (fig. S11, G and H). These data demonstrated that C188-9 and anti–PD-1 interact to augment antitumor responses in GBC.

## DISCUSSION

Despite advances in GBC diagnosis and treatment, the morbidity and mortality of this disease have not markedly decreased to date. Hence, the molecular mechanism underlying GBC occurrence and chemoresistance needs to be further investigated. In the present study, we reported that the IL-6R/JAK2/STAT3 axis transactivated HLF expression in GBC. We clarified that HLF promoted GBC CSCs’ self-renewal, tumorigenicity, and chemoresistance by inducing TFEB-mediated autophagy. Moreover, HLF/TFEB/PD-L1 axis determined the immune evasion of GBC cells. In addition, the IL-6R/p-STAT3/HLF/TFEB/PD-L1 axis was activated in GBC and associated with poor prognosis (Fig. 7I). These findings suggest that HLF might be a potential therapeutic target and personalized therapy biomarker for GBC.

Cholelithiasis is a cofactor in the development of GBC, and inflammatory factors play a vital role in this transformation process (*28*, *29*). Here, our single-cell transcriptome analysis observed that HLF levels were markedly up-regulated in cholelithiasis tissues and were enhanced upon *IL-6* stimulation. Accumulating evidence has illustrated that the IL-6/IL-6R/STAT3 pathway plays a pivotal role in tumor occurrence and progression (*30*); however, the detailed mechanism has not yet been identified. Using RNA-seq and CUT&Tag analyses, we found the promising result that *IL-6R* and *TFEB* are key downstream targets of HLF in GBC. Using multiple approaches, we confirmed that HLF directly binds to the *IL-6R* promoter and enhances IL-6R transcription and subsequent JAK2/STAT3 activation. Moreover, activated IL-6R/JAK2/STAT3 signaling up-regulated HLF expression, thus completing the HLF/IL-6R/JAK2/STAT3 regulatory circuit. The HLF-positive feedback loop augments TFEB activity to facilitate the CSC expansion, chemoresistance, and immune evasion of GBC cells. Considering the important role of the HLF/IL-6R/JAK2/STAT3 axis in GBC progression, we believe that targeting this axis could be a therapeutic strategy for GBC.

TFEB, a master regulator of autophagy, affects the degradation of misfolded proteins for proteostasis via trafficking, and, following translocation to the nucleus, transcriptionally regulates most lysosomal genes (*31*, *32*). TFEB overexpression induces lysosomal biogenesis and degradation via autophagy (*33*). Activation of *TFEB* has been reported to be involved in the regulation of several cancers. High TFEB expression is correlated with poor prognosis in glioblastoma, non–small-cell lung cancer (NSCLC), and breast cancer (*34*–*36*). However, its role in GBC remains unclear. Our current study revealed that a high level of TFEB was associated with poor OS in patients with GBC, and elevated TFEB levels promoted autophagy and the malignant properties of GBC cells. Moreover, TFEB is a bona fide target of HLF, and the promoting effects of HLF on GBC have been partially attributed to its regulatory effects on TFEB-mediated autophagy. More recently, the first direct TFEB inhibitor, eltrombopag, has been approved for the treatment of thrombocytopenia (*37*). Our findings support the oncogenic role of TFEB in GBC and suggest that TFEB is a potential new therapeutic target for GBC.

Gemcitabine is one of the main chemotherapeutic drugs for patients with advanced GBC (*5*, *38*). However, patients with GBC receiving gemcitabine have been shown to exhibit limited survival improvement due to the development of chemoresistance. Thus, it is urgent to elucidate the underlying mechanisms of chemoresistance and find reliable biomarkers that can predict chemotherapy response in patients with GBC. Here, we demonstrated that the HLF-overexpressing GBC cells were resistant to gemcitabine, and the HLF/TFEB/autophagy axis determines the gemcitabine response. More recently, a series of small-molecule compounds targeting autophagy has been developed (*39*). We also provide evidence for using an inhibitor of autophagy, which is the downstream effector of HLF, to restore gemcitabine response in HLF-mediated resistance. These results provide proof of concept that targeting autophagy is a promising strategy for overcoming gemcitabine resistance. Further studies using patient cohort, PDOs, and PDXs suggested that low HLF in GBCs was associated with superior survival in patients who received gemcitabine treatment. Therefore, evaluating HLF expression in patients with GBC to distinguish beneficiaries for gemcitabine therapy may be advisable and deserves further clinical trials.

PD-L1 and PD-1 play vital roles in tumor immune evasion; however, most patients with cancer are resistant to immune checkpoint blockade, and the underlying mechanisms of this resistance remain unclear. PD-L1 expression in cancer cells is regulated by a variety of transcriptional factors including hypoxia-inducible factor–1α, nuclear factor κB, STAT1, and MYC (*40*–*42*). Our findings revealed a strong association between the HLF/TFEB axis and PD-L1 protein in human tumors, where TFEB directly binds to the *PD-L1* promoter. We identified that HLF promotes immune evasion, which may explain its contribution to GBC development. Moreover, the enforced HLF expression was more sensitive to anti–PD-L1 immunotherapy in mice with GBC. This was further validated in GBC samples from patients, where tumor HLF and TFEB expression levels were associated with the response to anti–PD-L1 treatment. Therefore, the HLF/TFEB axis regulates inducible PD-L1 expression and may be associated with efficacy of PD-L1 checkpoint blockade.

The combination of immunotherapy and targeted therapy has achieved good results in human cancers (*43*, *44*). Inhibition of p-STAT3 with C188-9, which is currently undergoing clinical trials for cancer treatment (*45*), reduces PD-L1 expression levels in tumor cells, enhances CD8^+^ T cell infiltration, and inhibits tumor growth. Alleviation of these effects by systemic depletion of CD8^+^ T cells further supports the critical role of p-STAT3/HLF/TFEB transactivation–regulated PD-L1 expression in tumor immunogenicity. As expected, C188-9 combined with an anti–PD-1 antibody caused a more profound abrogation of immune checkpoint blockade, markedly enhanced CD8^+^ T cell infiltration, and blocked tumor growth. Therefore, patients with cancer will benefit from a combination of C188-9 and anti–PD-1 antibody therapy and warrant further clinical validation. Hence, our findings elucidate a new mechanism underlying PD-L1 up-regulation, which is controlled by the activated p-STAT3/HLF/TFEB axis and provides a molecular basis for improving the clinical response rate and efficacy of targeted therapy and PD-1/PD-L1 blockade therapy in patients with cancer.

Our study has some limitations. Despite finding that the HLF/TFEB axis drives immune evasion via PD-L1, the regulation of HLF/TFEB axis on tumor immunity may have other mechanisms. For example, the HLF/TFEB axis–mediated autophagy may not only support tumor cell survival and proliferation but also alter tumor antigen presentation, immune cell trafficking, and effector immune cell function in the tumor microenvironment (TME). More experiments are needed to confirm these hypotheses. Most of our findings are derived from preclinical mouse models and retrospective clinical cohorts. The predictive effect of HLF on chemotherapy and immunotherapy also needs to be further verified in human clinical trials.

In conclusion, our study highlighted the biological significance of HLF in gallbladder CSCs’ self-renewal, chemoresistance, and immune evasion. These findings demonstrate that HLF could act not only as a clinical biomarker for chemotherapy or immune checkpoint blockade but also as a therapeutic target for GBC.

## MATERIALS AND METHODS

### Patients with GBC and analysis

Eighty pairs of matched GBC and adjacent normal tissues were obtained from the Eastern Hepatobiliary Surgery Hospital (EHBH). GBC tumors and adjacent nontumor tissues from cohort 1 (*n* = 170) were obtained from patients undergoing surgical resection at the EHBH between 2013 and 2018. The detailed clinicopathological features of the patients in cohort 1 are described in table S1. Cohort 2 (*n* = 209) consisted of patients who underwent surgical resection of primary GBC at the Fudan University Shanghai Cancer Center, Shanghai General Hospital, and Shanghai East Hospital between 2012 and 2023 (table S2). OS was defined as the time from surgery to death. “Liver metastasis” was defined as perihepatic liver metastasis.

### Gemcitabine therapy cohort

Cohort 3 (*n* = 60) consisted of patients with GBC who received gemcitabine after cholecystectomy for EHBH between 2019 and 2021. The detailed clinicopathological features are described in table S3. Gemcitabine was administered to the patients at 1000 mg/m^2^ on days 1 and 8, repeated every 3 weeks.

### Immune therapy cohort

Cohort 4 (*n* = 30) consisted of patients with GBC who received anti–PD-L1 after cholecystectomy at the EHBH between 2018 and 2020 (table S4). Camrelizumab, a kind of PD-L1 inhibitor, was then initiated with 200 mg intravenously every 3 weeks.

The above study protocols were approved by the ethics committee of the EHBH, Naval Military Medical University (no. EHBHKY2020-K-016). Informed consent was obtained from all the participants included in this study according to the committee regulations.

### Cell lines and lentivirus

The GBC cell lines—SGC-996, NOZ, and GBC-SD—were obtained from the Cell Bank of the Type Culture Collection of the Chinese Academy of Sciences (the Shanghai Institute of Cell Biology of the Chinese Academy of Sciences). The GBC cell lines were cultured in Dulbecco’s modified Eagle’s medium (DMEM; Gibco). DMEM was supplemented with 10% fetal bovine serum (FBS; Gibco), penicillin (100 IU/ml; Gibco), and streptomycin (100 μg/ml; Gibco). All the cells were maintained in a humidified 5% CO_2_ incubator at 37°C. *HLF* knockdown, *TFEB* knockdown, *IL-6R* knockdown, HLF, TFEB, IL-6R overexpression, and control lentiviruses were purchased from ObiO Technology Co. (Shanghai, China). An anti-Flag antibody was used to recognize exogenous Flag-tagged HLF proteins in the Flag-tagged HLF-infected cells.

### Preparation and cultivation of primary GBC cells

The experimental procedures were performed according to the guidelines of the charitable state-controlled foundation Human Tissue and Cell Research, with the informed patient’s consent approved by the local ethical committee of the EHBH. Briefly, fresh GBC tumors were collected from patients with GBC undergoing cholecystectomy. The fresh tissues were followed by 60 min of incubation with collagenase IV (10 mg/ml; Sigma-Aldrich) and trypsin (0.10%; Sigma-Aldrich) at 37°C. The specimens were filtered through a sterile 100-mm strainer. Red blood cells were eliminated with a hypoosmotic red blood cell lysis buffer (eBioscience). The freshly isolated primary GBC cells were maintained on Matrigel-coated culture plates (Merck) and cultured in RPMI 1640 medium supplemented with 2 mM glutamine (Invitrogen), 20% FBS (Gibco), 1 μM dexamethasone (Sigma-Aldrich), human epidermal growth factor (EGF) (40ng/ml; PeproTech), human fibroblast growth factor (FGF) (20 ng/ml; PeproTech), human insulin (5 μg/ml; Sigma-Aldrich), penicillin (100 IU/ml; Gibco), and streptomycin (100 μg/ml; Gibco).

### Spheroid assay

Three hundred single cells were seeded into 96-well Ultra-Low Attachment Microplates (Corning, USA) in serum-free DMEM/F12 (Invitrogen, USA), supplemented with B27 (1:50; Invitrogen), EGF (20 ng/ml; PeproTech), basic FGF (10 ng/ml; Invitrogen), and insulin (4 mg/ml; Sigma-Aldrich). Spheres were photographed and counted 7 days after seeding (primary spheres).

### In vitro limiting dilution assay

GBC cells were seeded into 96-well ultra-low attachment culture plates at various cell numbers and incubated for 7 days. On the basis of the frequency of wells with sphere forming, the proportion of CSCs was determined using Poisson distribution statistics and the LCalc version 1.1 software program (Stem Cell Technologies Inc., Vancouver, Canada). Briefly, limiting dilution analysis (LDA) accepts an input data table of three or four columns, separated by any combination of commas, spaces, or tabs. The data can be directly type into the webpage text field or can simply cut and paste the whole table from any spreadsheet application. Each row of data gives results for a particular cell dose. The columns are as follows: (i) dose: number of cells in each culture, (ii) tested: number of cultures tested, (iii) response: number of positive cultures, and (iv) group (optional): label for the population group to which cells belong. By default, Extreme Limiting Dilution Assay (ELDA) computes a 95% confidence for the active cell frequency in each population group.

### Flow cytometric analysis

The apoptosis of hepatoma cells was measured by flow cytometry using a Fluorescein Isothiocyanate (FITC) Annexin V Apoptosis Detection Kit (51-66211E, BD Biosciences). Briefly, 1 × 10^6^ cells were harvested and washed twice with cold cell staining buffer, resuspended in 100 μl of annexin V binding buffer, and then incubated with 5 μl of FITC annexin V and 5 μl of propidium iodide (PI) viability staining solution for 15 min at room temperature in the dark. The cell suspension was then incubated with 400 μl of annexin V binding buffer, followed by flow cytometry analysis.

### Cell survival assay

GBC cells were seeded at a concentration of 5000 cells per well in 96-well plates and equilibrated at 37°C and 5% CO_2_ for 12 hours. Then, GBC cells were treated with indicated concentrations of gemcitabine [dissolved in dimethyl sulfoxide (DMSO), drug concentrations ranged from 0.01 to 1000 μM, DMSO used as a negative control, and *n* = 4 for each concentration] for 48 hours. Cell viability was quantified using WST-1 reagent (Roche) according to the manufacturer’s instructions. All experiments were performed in triplicate.

### Tissue dissociation and organoid culture

Organoid culture was performed as previously described (*46*). Fresh GBC tissue was obtained; the blood, fat, necrotic, and connective tissue on the tissue were cut off; and the area with abundant tumor cells was preserved and cut into pieces. The tissues were put into 5 ml of 5 mM phosphate-buffered saline (PBS)/EDTA liquid for 15 min at room temperature. Then, the tissues were placed in 5 ml of 1 mM PBS/EDTA containing 2× TrypLE and digested at 37°C for 1 hour. The cells were suspended in liquid by mechanical force blowing away the tissue mass. Dissociated cell clusters were spun down at 350 g and resuspended with 60% Matrigel/organoid culture medium. A drop of 250 μl of resuspension was plated in one well of a six-well plate coated with 60% Matrigel and incubated at 37°C and 5% CO_2_ for 30 min. After the drops became solid, 1.5 ml of the organoid culture medium was added to the well, and the medium was changed every 72 hours.

### Organoid drug response assay

To plate organoids for analyses of drug response, organoids were collected 4 to 5 days after passaging and passed through a 100-μm cell strainer (Corning no. 352360) to eliminate large organoids. Subsequently, organoids were resuspended in 2% Matrigel/organoid culture medium (15,000 to 20,000 organoids/ml) and dispensed into ultralow-attachment 96-well plates (Corning) in triplicate. At 24 hours after plating, gemcitabine was added at various concentrations (ranged from 0.01 to 100 μM). The vehicle (DMSO; D8418, Sigma-Aldrich) was used as a negative control. Cell viability was assayed using CellTiter-Glo 3D (Promega) according to the manufacturer’s instructions following 7 days of drug incubation, and results were normalized to vehicle controls.

### In vivo limiting dilution assay

For the in vivo limiting dilution assay, GBC cells were digested into single cells and counted. The indicated numbers of cells (1 × 10^3^, 5 × 10^3^, 1 × 10^4^, and 5 × 10^4^) were mixed with Matrigel (1:1) and subcutaneously injected into NOD-SCID mice (*n* = 8 each group). CQ (Sigma-Aldrich; 25 mg/kg) was injected intraperitoneally once daily for 1 week.

### Patient-derived xenografts

BALB/cNj-Foxn1nu/Gpt (BALB/c) nude mice (male and eight weeks old) and huCD34^+^HSC-NCG (huHSC-NCG) mice were purchased from GemPharmatech Co. Ltd. (Jiangsu, China). Mice were maintained at a 12-hour/12-hour dark-light cycle, ~25°C, and ~60% humidity and given food and water ad libitum. The mice were assigned to each group by random allocation.

For the PDX model, primary tumor samples were obtained for xenograft establishment as described previously. When the PDX volume reaches ~150 mm^3^, BALB/c nude mice were randomly assigned to treatment with vehicle or gemcitabine (50 mg/kg; intraperitoneal injection twice a week) for a total of 4 weeks (*n* = 5 each group). huHSC-NCG mice were injected intraperitoneally every 4 days with atezolizumab (10 mg/kg) or human immunoglobulin G1 (IgG1) control (BE0297, Bio X Cell) for a total of 3 weeks (*n* = 4 each group). Tumor volumes were measured by caliper twice a week using the formula volume = (width^2^ × length) × 0.5, where *L* is the longest tumor axis and *W* is the shortest tumor axis. Mice were euthanized by CO_2_, and the tumor was sectioned or frozen for the following analysis.

### Mouse xenografts

NOZ short hairpin HLF (shHLF) and control cells (1 × 10^6^ cells per mouse) were injected subcutaneously into the right posterior flanks of 8-week-old BALB/c nude mice. When the xenograft volume reaches ~150 mm^3^, BALB/c nude mice were randomly assigned to treatment with vehicle or gemcitabine (50 mg/kg; intraperitoneal injection twice a week) for a total of 4 weeks (*n* = 5 each group).

NOZ HLF and control cells (1 × 10^6^ cells per mouse) were injected subcutaneously into the right posterior flanks of huHSC-NCG mice. On day 3 after inoculation, mice were injected intraperitoneally every 4 days with atezolizumab (10 mg/kg) or human IgG control (*n* = 5 each group). NOZ cells (1 × 10^6^ cells per mouse) were injected subcutaneously into the right posterior flanks of huHSC-NCG mice. On day 3 after inoculation, mice were injected intraperitoneally with C188-9 (100 mg/kg; S8605, Selleck) or vehicle five times a week. In addition, mice were injected intraperitoneally every 4 days with antihuman PD-1 (with 10 mg/kg; pembrolizumab, Bio X Cell) or human IgG4 control (*n* = 5 each group).

Tumor volumes were measured by caliper twice a week using the formula volume = (width^2^ × length) × 0.5, where *L* is the longest tumor axis and *W* is the shortest tumor axis. Mice were euthanized by CO_2_, and tumor was sectioned or frozen for the following analysis. All procedures performed in studies involving animals were in accordance with the ethical standards of the institutional and/or national research committee and with the 1964 Helsinki declaration and its later amendments or comparable ethical standards. All procedures and protocols were approved by the ethics committee of EHBH. All animal experiments and relevant details were conducted in accordance with the approved guidelines and were approved by the committee on Animal Care and Use of Naval Military Medical University (no. EDWLL-002-1).

### ChIP assay

ChIP assay was carried out in NOZ and SGC-996 cells using an EpiTect ChIP qPCR Kit (QIAGEN) as described previously. Briefly, 1 × 10^7^ cells fixed with formaldehyde were collected and added with 500 μl of lysis buffer. Then, lysate was sonicated for 25 cycles of 6-s power-on and 30-s interval with an intensity of 200 W. Next, the supernatants were mixed with Protein A/G magnetic beads. Then, chromatin was immunoprecipitated with IgG or anti-Flag, anti-STAT3, and anti-TFEB antibodies overnight. The next day, the mixture was washed and incubated with elution buffer at 62°C for 2 hours and then at 95°C for 10 min. Then, bound DNA was purified and complied to qPCR.

### Dual-luciferase reporter gene assay

The DNA sequences containing the promoter region of HLF (−1550 to +56), IL-6R (−1600 to +56), TFEB (−1900 to +56), and PD-L1 (−1950 to +56) were cloned into pGL6-luc plasmid (wild-type plasmids, designated as HLF-WT, IL-6R-WT, TFEB-WT, and PD-L1-WT). The potential HLF-binding sites within the IL-6R or TFEB promoter, the potential STAT3-binding sites within the HLF promoter, and the potential TFEB-binding sites within the PD-L1 promoter were further truncated or mutated in pGL6-IL-6R-WT-luc, pGL6-TFEB-WT-luc plasmid, pGL6-HLF-WT-luc plasmid, and pGL6-PD-L1-WT-luc, respectively (mutant plasmids). The cells were transfected with one of the above plasmids (wild-type plasmids or mutant plasmids) and pRL-TK-Renilla-luc plasmids. For TOP/FOP flash reporter assay, the TOPflash reporter plasmids, containing wild-type T cell factor (TCF) receptor, and FOPflash reporter plasmids, containing mutant TCF receptor, were obtained from Promega. Cells were cotransfected with TOPflash or FOPflash and pRL-TK-Renilla-luc plasmid. The luciferase reporter gene assays were performed as previously described. Briefly, firefly luciferase activity of each group in triplicate was analyzed by Dual-Luciferase Reporter Assay Kit (Promega) using a Synergy 2 Multidetection Microplate Reader (BioTek Instruments). Firefly luciferase activity was normalized to Renilla luciferase activity.

### Western blotting assays

Protein extracts of cell lysate or human GBC samples were subjected to SDS–polyacrylamide gel electrophoresis and then transferred to the nitrocellulose membrane. The protein band was incubated with primary antibodies and IRDye 800CW–conjugated second antibody on the LI-COR imaging system (LI-COR Biosciences). The antibodies used were listed in table S5.

### Real-time qPCR

Total RNA was extracted from tissues or cells using TRIzol (Invitrogen) and was reverse transcribed using a Reverse Transcription System (Promega) to synthesize cDNA. In the Roche Light Cycler 96 System (Roche, USA), the cDNA was mixed with SYBR Green PCR Kit (Roche) and specific primers to actuate real-time PCR. PCR conditions included 1 cycle at 95°C for 5 min, followed by up to 40 cycles of 95°C for 15 s (denaturation), 60°C for 30 s (annealing), and 72°C for 30 s (extension). The specificity of primers was confirmed by melting curves following the reaction. Each sample was measured in triplicate biological replicates. Each experiment was repeated at least three times, and the representative results were shown. The sequences for primers are listed in table S6.

### IHC staining

IHC staining was described previously. Briefly, tissue samples or xenografts were formalin fixed and paraffin embedded (FFPE) and sectioned for the following IHC examination. Sections were deparaffinized in dimethyl benzene and a ladder concentration of ethanol (100, 95, 85, and 75%), heated for antigen retrieval, blocked by 3% H_2_O_2_ and 1% bovine serum albumin, incubated with primary antibody and secondary antibody, and colored with diaminobenzidine. The stained sections were scanned using the ScanScope XT scanner (Aperio Technologies Inc.). The quality of immunostaining was independently evaluated by two pathologists blindly. The high-resolution digital images were scored by a pathologist using “positive pixel count v9” algorithms provided by the ImageScope program (Aperio Technologies Inc.). Briefly, the program automatically counted pixels and measured the intensity of positive (brown staining) pixels. The staining score for each sample was defined as the average positivity (sum intensity of positive pixels per total positive pixels) times positivity (number of positive pixels per total pixels). The median HLF, IL-6R, p-STAT3, TFEB, and PD-L1 score was set as the cutoff value to divide the cohort into high and low subgroups.

Immunofluorescent staining was performed using a tyramide signal amplification (TSA) fluorescence kit (TSA Plus Fluorescein, NEL741001KT, PerkinElmer, Waltham, Massachusetts, USA) according to the manufacturer’s instructions. The antibodies used for IHC or immunofluorescent staining are listed in table S5.

### Immunofluorescence staining

Cells were fixed with 4% formalin for 15 min at room temperature. After being incubated with 0.1% Triton X-100 for 5 min, cells were blocked for 1 hour. Cells were incubated with primary antibody at 4°C overnight and secondary antibody (Goat anti-Mouse IgG Alexa Fluor 488 and Goat anti-Rabbit IgG Alexa Fluor 594 from Life Technologies) for 30 min at room temperature. Cells were then stained with 4′,6-diamidino-2-phenylindole (DAPI; Sigma-Aldrich) and observed under a fluorescent/confocal microscope (Olympus).

### CUT&Tag library and sequencing

According to the protocol, the CUT&Tag library was constructed using Hyperactive In-Situ ChIP Library Prep Kit (TD901, Vazyme, China). In short, cells were harvested, counted, and centrifuged for 3 min at 600*g* at room temperature. Aliquots of cells (60,000 to 500,000 cells) were washed twice in 500 μl of wash buffer by gentle pipetting. Concanavalin A (Con A)–coated magnetic beads were prepared as the kit manual described, and 10 μl of activated beads were added per sample and incubated at room temperature for 10 min. According to the kit instructions, cells were sequentially incubated with Con A beads, primary antibody, secondary antibody, and hyperactive PG-TN5/PA-TN5 transposon and then fragmented. The fragmented DNA was extracted from the samples, and the extracted DNA was amplified by PCR to obtain the library, which was sequenced using PE150 by the Illumina sequencer (Shanghai OE Biotech, China). Sequencing data have been deposited at the National Center for Biotechnology Information (NCBI) BioProject under accession number PRJNA1142754.

### Data processing for CUT&Tag

Fastp v0.20.0 was used to remove adapters and low-quality reads. Paired-end reads were aligned using Bowtie2 version 2.3.4.3 with the option -end-to-end-sensitive. Duplicated reads are removed using Picard v2.18.17 with this parameter: REMOVE_DUPLICATES = true. Peak calling uses SEACR v1.3 with a threshold of 0.01. Scatterplots, correlation plots, and heatmaps are displayed using deepTools v2.27.1. Annotation of peaks is performed using an R package ChIPseeker v1.12.1. MEME-ChIP v5.0.5 was used to search for the binding site.

### RNA sequencing

Total RNA was extracted using the mirVana miRNA Isolation Kit (Ambion) following the manufacturer’s protocol. RNA integrity was evaluated using the Agilent 2100 Bioanalyzer (Agilent Technologies, Santa Clara, CA, USA). The samples with RNA integrity number ≥ 7 were subjected to the subsequent analysis. The libraries were constructed using TruSeq Stranded mRNA Sample Prep Kit (Illumina, San Diego, CA, USA) according to the manufacturer’s instructions. Then, these libraries were sequenced on the Illumina sequencing platform (HiSeq 2500 or Illumina HiSeq X Ten), and 125/150–base pair paired-end reads were generated.

The transcriptome sequencing and analysis were conducted by OE Biotech Co. Ltd. (Shanghai, China). Raw data (raw reads) were processed using Trimmomatic. The reads containing poly-*N* and the low-quality reads were removed to obtain the clean reads. Then, the clean reads were mapped to the reference genome using HISAT2. The fragments per kilobase of exon model per million mapped fragments (FPKM) value of each gene was calculated using cufflinks, and the read counts of each gene were obtained by htseq-count. Differential expression analyses (DEGs) were identified using the DESeq (2012) R package functions estimate size factors and nbinom test. *P* < 0.05 and fold change > 2 or < 0.5 were set as the threshold for significant differential expression. Hierarchical cluster analysis of DEGs was performed to explore the gene expression pattern. GO and gene set enrichment analysis of DEGs were respectively performed using R based on the hypergeometric distribution. Sequencing data have been deposited at the NCBI BioProject under accession numbers PRJNA1142594 and PRJNA1142756.

### Single-cell transcriptomic data analysis

#### Cell clustering and annotation

We reanalyzed published single-cell RNA-seq datasets from patients with adenomyomatosis, cholelithiasis, and gallbladder carcinoma. Filtered gene expression matrices were processed using the Seurat package (v5.2.1) in R (v4.3.2). Initial quality control included the following: retention of genes detected in >0.1% of cells, exclusion of low-quality cells meeting either criterion of <200 detected genes, and >20% mitochondrial unique molecular identifiers (UMIs). Data normalization was performed via SCTransform (v0.4.1), followed by batch effect correction using Harmony (v1.2.0). UMAP dimensionality reduction (30 principal components) and cell clustering (resolution = 0.5) were implemented for initial population identification. Minor subclusters coexpressing two or more canonical markers were identified as potential doublets and manually excluded from downstream analyses.

Cell types were annotated using the following canonical markers: epithelial cells: EPCAM and Keratin 19 (KRT19); endothelial cells: PECAM1, CD34, and Willebrand factor (VWF); stromal cells: COL1A1, lumican (LUM), and regulator of G-protein signaling 5 (RGS5); mast cells: KIT, Carboxypeptidase A3 (CPA3), and TPSAB1; myeloid cells: CD14, CD68, and CD163; B cells: CD79A and membrane spanning 4-domains A1 (MS4A1); plasma cells: CD79A and MZB1; T cells: CD2, CD3D, and CD3E. Epithelial subclustering was performed using similar preprocessing parameters but with an increased clustering resolution (0.8).

#### Cellular stemness analysis

Cellular stemness potential was quantified using CytoTRACE (v0.3.3) with default parameters. Visualization of stemness scores across epithelial subpopulations was achieved through density plots (scCustomize::Plot_Density_Custom, v3.0.1) and ridge plots (Seurat::RidgePlot).

#### GSVA enrichment analysis

GSVA (v1.50.0) was applied to epithelial subclusters 0, 5, and 7 (high-stemness populations) using the HALLMARK gene sets from msigdbr packages (7.5.1). Enrichment patterns were visualized as a clustered heatmap via pheatmap (v1.0.12), with row-wise *z*-score normalization of pathway activity scores.

### T cell–mediated tumor cell killing assay

Human PBMCs were cultured in RPMI 1640 medium and activated with Dynabeads Human T-Activator CD3/CD28 (Gibco, CA, USA) for 1 week according to the manufacturer’s instructions. GBC cells were seeded into 12-well plates at a cell-dependent concentration. After 24 hours, activated PBMCs were cocultured with adhered GBC cells for 48 hours at a ratio of 3:1. After 48 hours of incubation, cell debris was removed, PBMCs were collected, and GBC cells were harvested and labeled with annexin V and PI for fluorescence-activated cell sorting (FACS) analysis.

### Coculture and expression of interferon-γ and IL-2

GBC cells were seeded in six-well plates (2 × 10^5^ cells per well) and cultured overnight. Then, PBMCs were added to wells at a ratio of 4:1 with respect to attached GBC cells. After coculture for 72 hours, GBC cells were harvested and analyzed for apoptosis based on FACS of cells stained with PI (Invitrogen) and the human annexin V–FITC kit (Invitrogen) according to the manufacturer’s protocol. PBMCs were harvested from the coculture system and stained with antibodies against human CD3, CD8, and interferon-γ (IFN-γ; BD Pharmingen). Supernatant from the cocultures was centrifuged at 14,000 rpm and subjected to IFN-γ and IL-2 analysis using an enzyme-linked immunosorbent assay (ELISA) kit (R&D Systems).

### Data analysis

Statistical analyses were performed using the SPSS software (version 22.0; SPSS Inc.). The data are expressed as the mean ± SD, and sample sizes are indicated in the figure legends. For all the experiments, more than three biological replicates were performed under the same conditions. Representative results from at least two independent experiments are shown. Student’s *t* test was used to compare two variables. Multivariate analysis was performed using the Cox multivariate proportional hazard regression model in a stepwise manner (forward: likelihood ratio). The relationships between the two variables were analyzed using the Pearson correlation method. A two-sided *P* value less than 0.05 was considered statistically significant.
