# Supplementary material for: HLF transactivates TFEB to promote gallbladder cancer stem cells’ self-renewal and determines tumor response to distinct therapies
Source: Sci Adv. 2025 Aug 8;11(32):eadv6723. doi: 10.1126/sciadv.adv6723 (PMC12333681; doi:10.1126/sciadv.adv6723)
Supplement: Supplementary file 1 — Figs. S1 to S11 Tables S1 to S6 [file sciadv.adv6723_sm.pdf]

Science Advances
AAAS

Supplementary Materials for

HLF transactivates *TFEB* to promote gallbladder cancer stem cells' self-renewal and determines tumor response to distinct therapies

Daimin Xiang *et al.*

Corresponding author: Jing Fu, fujing-724@163.com; Hongyang Wang, hywangk@vip.sina.com

Sci. Adv. **11**, eadv6723 (2025)
DOI: 10.1126/sciadv.adv6723

This PDF file includes:

Figs. S1 to S11
Tables S1 to S6

fig. S1

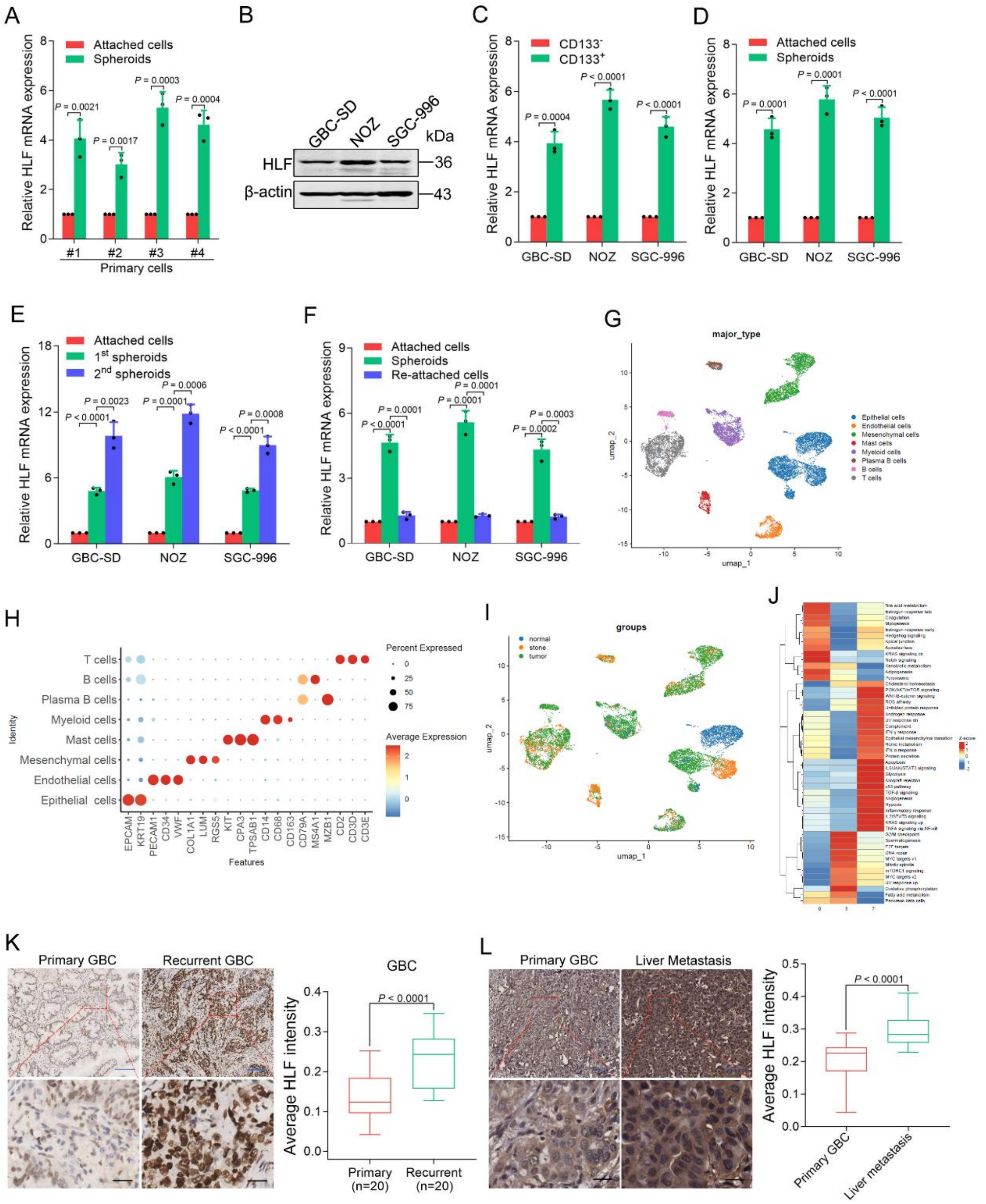

fig. S1 HLF expression is upregulated in gallbladder CSCs.

(A) Real-time PCR analysis of HLF expression in primary GBC adherent cells and spheres (n = 3).

(B) Western blot analysis of HLF expression in GBC cell lines.

(C) Real-time PCR analysis HLF expression in sorted CD133^{+} GBC cell relative to negative cells (n = 3).

(D) Real-time PCR analysis of HLF expression in GBC adherent cells and spheres (n = 3).

(E) Real-time PCR analysis of HLF expression in serial passages of GBC spheroids (n = 3).

(F) Real-time PCR analysis of HLF expression in GBC adherent, spheroids, and re-adherent cells (n = 3).

(G) UMAP plot showing 13,359 cells of three patients, annotated by cell types.

(H) Bubble plot showing marker genes expression of 8 cell types.

(I) UMAP plot shows sample source of all cells.
(J) Gene Set Variation Analysis (GSVA) the HALLMARK gene sets from MSigDB to investigate downstream regulatory pathways in three HLF-high subclusters (clusters 0, 5, and 7).
(K) Representative images of IHC staining of HLF in recurrent GBCs and primary lesions of patient. Blue scale bar = 5 μm. Black scale bar = 25 μm.
(L) Representative images of IHC staining of HLF in liver metastatic tissues and primary GBC lesions of patient (n = 20). Blue scale bar = 5 μm. Black scale bar = 25 μm.

fig. S2

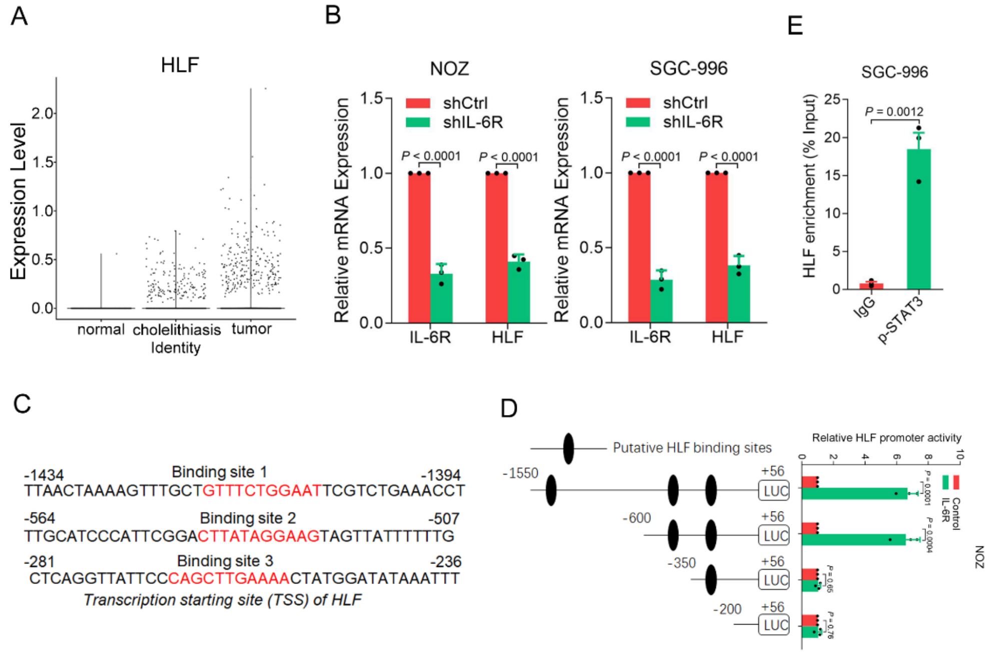

fig. S2 IL-6/IL-6R/STAT3 axis upregulates HLF expression in GBC cells.

(A) Violin plot of the expression of HLF (normal, cholelithiasis and GBC tissues).

(B) Real-time polymerase chain reaction (PCR) analysis of the mRNA expression of HLF and IL-6R in shIL-6R or control GBC cells (n = 3).

(C) Potential binding sites of p-STAT3 on HLF promoter.

(D) Deletion analysis identified p-STAT3-responsive regions in the *HLF* promoter. Serially truncated HLF promoter constructs were transfected into NOZ IL-6R overexpressing and control cells, and relative luciferase activities were determined (n = 3).

(E) SGC-996 cells were subjected to ChIP assay with anti-STAT3(Y705) or anti-IgG antibody followed by real-time PCR (n = 3).

fig. S3

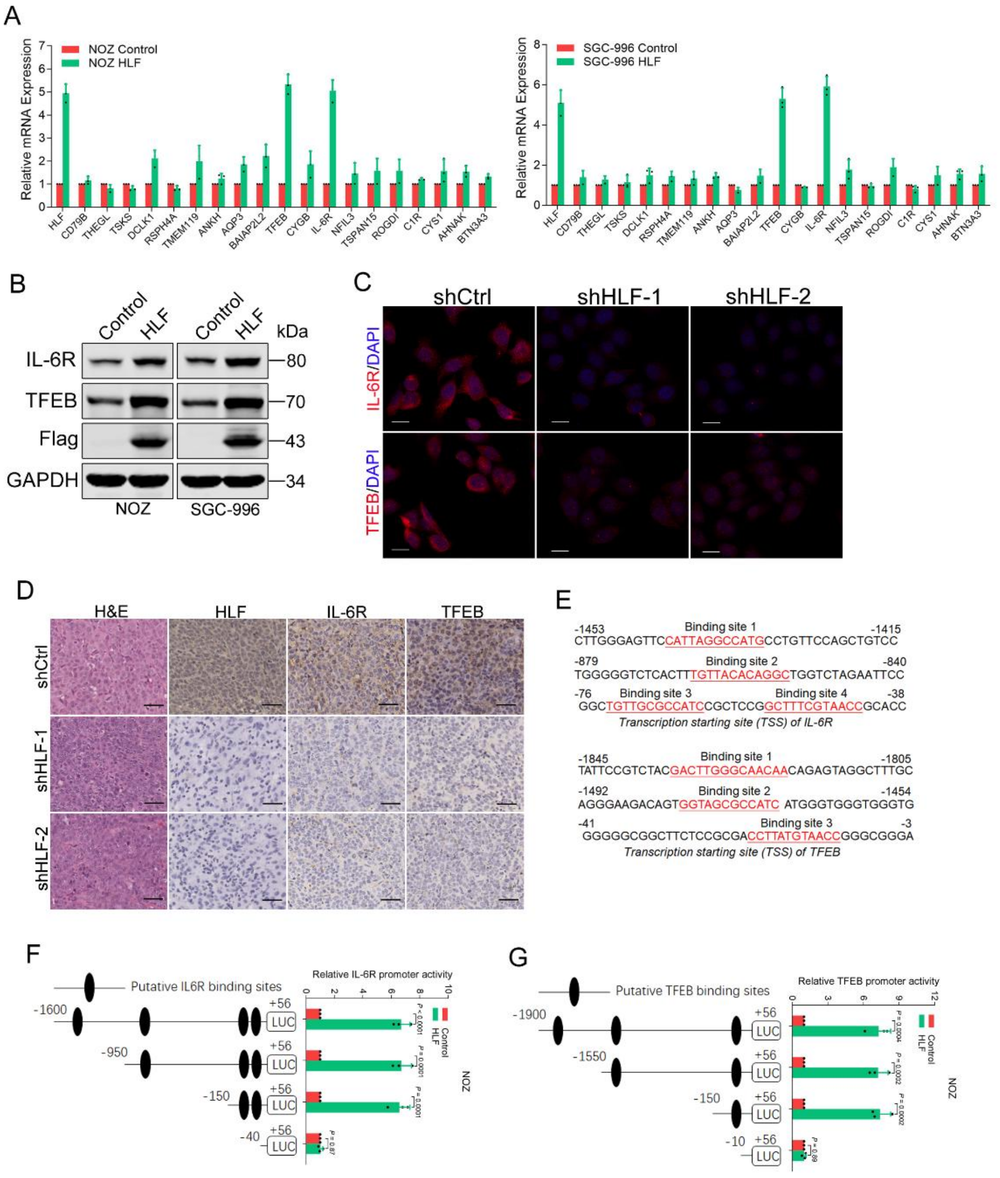

fig. S3 HLF increases IL-6R and TFEB expression in GBC cells.

(A) Real-time PCR analysis of the mRNA expression of potential targeted genes in HLF overexpression or control NOZ/SGC-996 cells (n = 3).

(B) Western blot analysis of the protein expression of HLF, IL-6R and TFEB in HLF overexpression or control GBC cells.

(C) Representative images of dual immunofluorescence staining of IL-6R and TFEB in HuCCT1 shHLF or control cells. The nuclei were counterstained with DAPI. Scale bar = 20 μm.

(D) Representative images of hematoxylin and eosin (H&E) and IHC staining of HLF, IL-6R and TFEB in xenografted tumors formed by NOZ shHLF or control cells. Scale bar = 25 μm.

(E) Potential binding sites of HLF on IL-6R and TFEB promoter.

(F) Deletion analysis identified HLF-responsive regions in the IL-6R promoter. Serially truncated IL-6R promoter constructs were transfected into NOZ HLF overexpressing and control cells, and relative luciferase activities were determined (n = 3).

(G) Deletion analysis identified HLF-responsive regions in the TFEB promoter. Serially truncated

TFEB promoter constructs were transfected into NOZ HLF overexpressing and control cells, and relative luciferase activities were determined (n = 3).

fig. S4

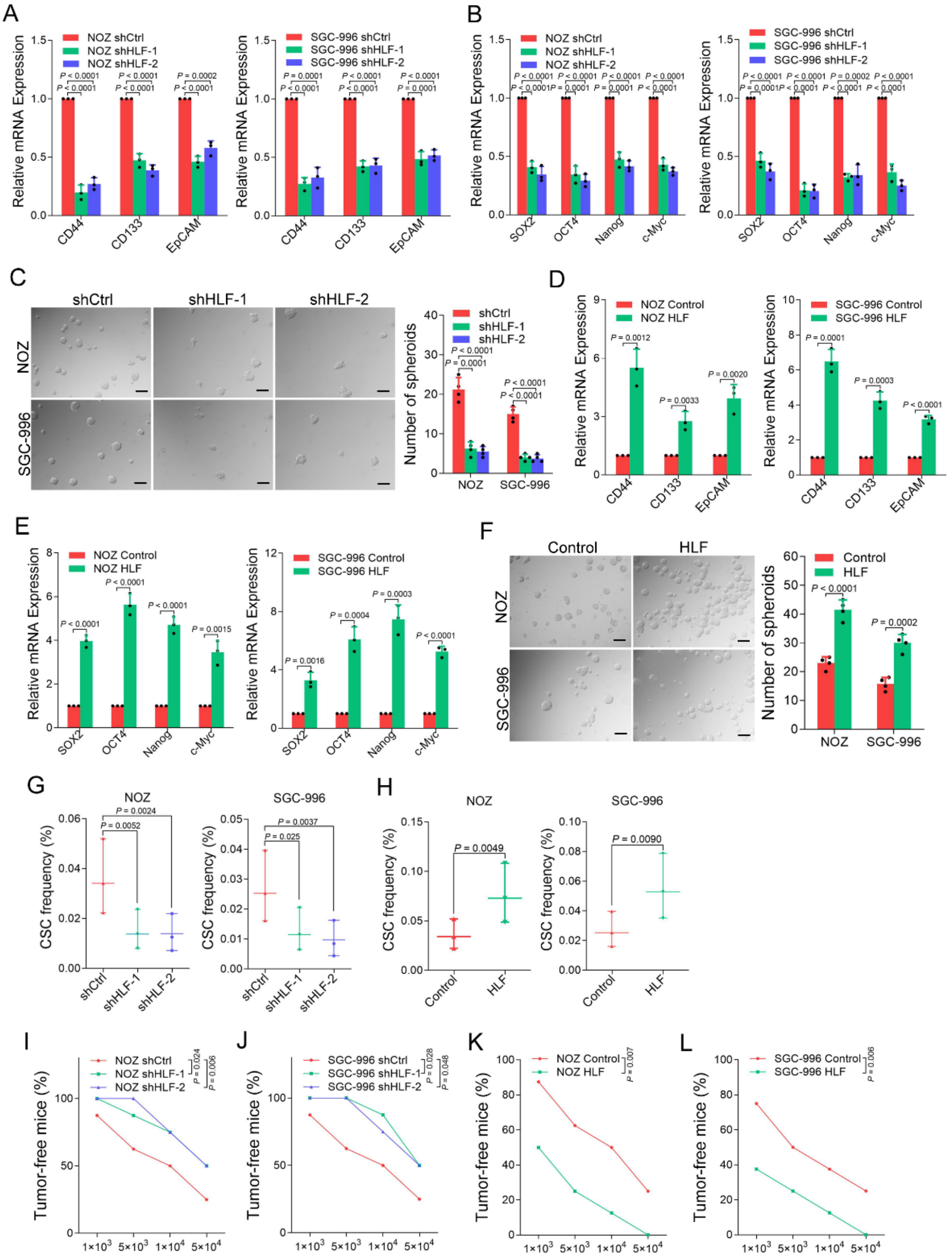

fig. S4 HLF facilitates gallbladder CSCs self-renewal.

(A) Real-time PCR analysis of CSC markers in NOZ/SGC-996 shHLF cells and control cells (n = 3).

(B) Real-time PCR analysis of pluripotent transcription factors in NOZ/SGC-996 shHLF cells and control cells (n = 3).

(C) Representative images of spheroids generated from shHLF GBC cells and control cells. The number of spheroids was counted and compared (n = 4). Scale bar = 100 µm.

(D) Real-time PCR analysis of CSC markers in HLF overexpression and control GBC cells (n = 3).

E) Real-time PCR analysis of pluripotent transcription factors in HLF overexpression and control GBC cells (n = 3).

(F) Representative images of spheroids generated from HLF overexpression and control GBC cells.

The number of spheroids was counted and compared (n = 4). Scale bar = 100 μm.

(G) The frequency of gallbladder CSCs in shHLF GBC cells and control cells was compared by *in vitro* limiting dilution assay.

(H) The frequency of gallbladder CSCs in HLF overexpression and control GBC cells was compared by *in vitro* limiting dilution assay.

(I) NOZ shHLF and control GBC cells were inoculated into NOD-SCID mice subcutaneously (n = 8), and the tumorigenicity was evaluated two months post inoculation.

(J) SGC-996 shHLF and control GBC cells were inoculated into NOD-SCID mice subcutaneously (n = 8), and the tumorigenicity was evaluated one month post inoculation.

(K) NOZ HLF and control GBC cells were inoculated into NOD-SCID mice subcutaneously (n = 8), and the tumorigenicity was evaluated two months post inoculation.

(L) SGC-996 HLF and control GBC cells were inoculated into NOD-SCID mice subcutaneously (n = 8), and the tumorigenicity was evaluated one month post inoculation.

fig. S5

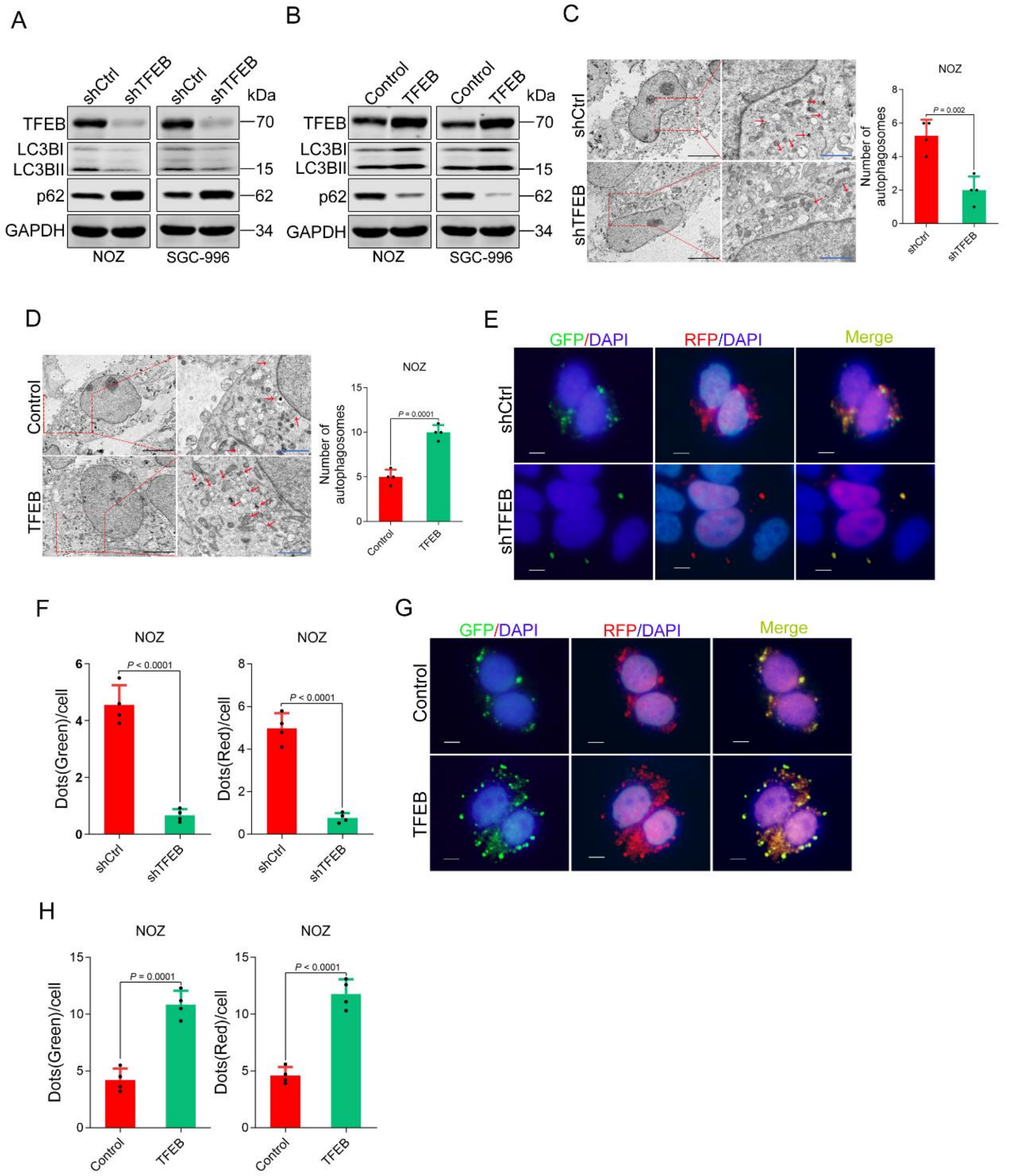

fig. S5 TFEB promotes GBC cells autophagy.

(A) Western blot analysis of LC3 expression and p62 expression in shTFEB or control GBC cells.

(B) Western blot analysis of LC3 expression and p62 expression in TFEB overexpression or control GBC cells.

(C) Autophagosomes were observed in shTFEB or control GBC cells under the transmission electron microscopy (TEM). Yellow arrows indicate the autophagosomes. Blue scale bar = 2 μm. Black scale bar = 5 μm.

(D) Autophagosomes were observed in TFEB overexpression or control GBC cells under the transmission electron microscopy (TEM). Yellow arrows indicate the autophagosomes. Blue scale bar = 2 μm. Black scale bar = 5 μm.

(E and F) GBC cells expressing mRFP-EGFP-LC3 were transfected with siNC and siTFEB. Autophagosomes were observed under a confocal microscope. Scale bar = 5 μm.

(G and H) GBC cells expressing mRFP-EGFP-LC3 were transfected with PCL3.1 and PCL3.1-

TFEB. Autophagosomes were observed under a confocal microscope. Scale bar = 5 μm.

fig. S6

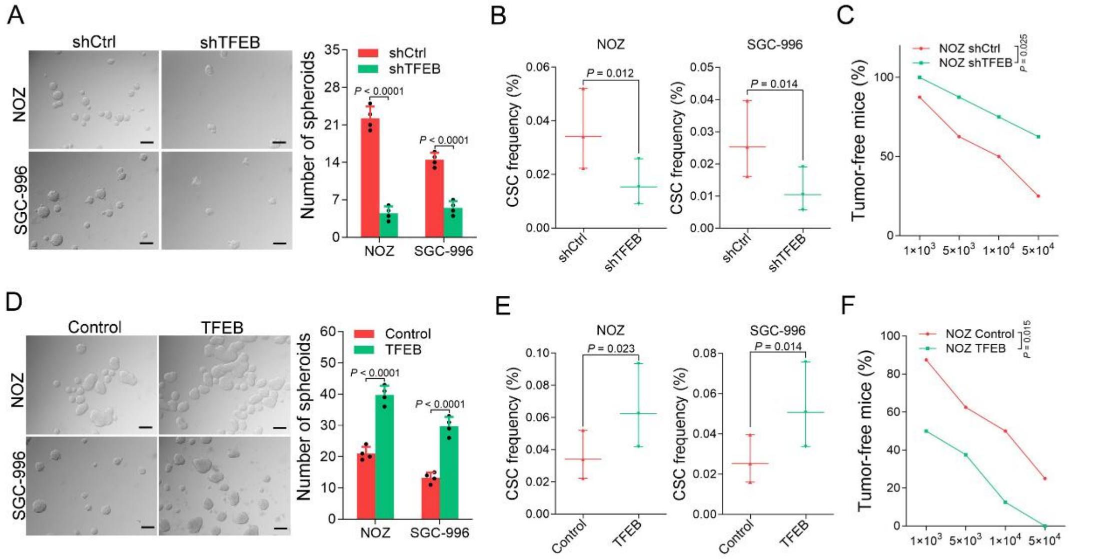

fig. S6 TFEB promotes GBC CSCs self-renewal.

(A) Representative images of spheroids generated from shTFEB GBC cells and control cells. The number of spheroids was counted and compared (n = 4). Scale bar = 100 μm.

(B) The frequency of gallbladder CSCs in shTFEB GBC cells and control cells was compared by *in vitro* limiting dilution assay.

(C) NOZ shTFEB or control cells were inoculated into NOD-SCID mice subcutaneously (n = 8), and the tumorigenicity was evaluated two months post inoculation.

(D) Representative images of spheroids generated from TFEB overexpression and control GBC cells. The number of spheroids was counted and compared (n = 4). Scale bar = 100 μm.

(E) The frequency of gallbladder CSCs in TFEB overexpression and control GBC cells was compared by *in vitro* limiting dilution assay.

(F) NOZ TFEB or control cells were inoculated into NOD-SCID mice subcutaneously (n = 8), and the tumorigenicity was evaluated two months post inoculation.

fig. S7

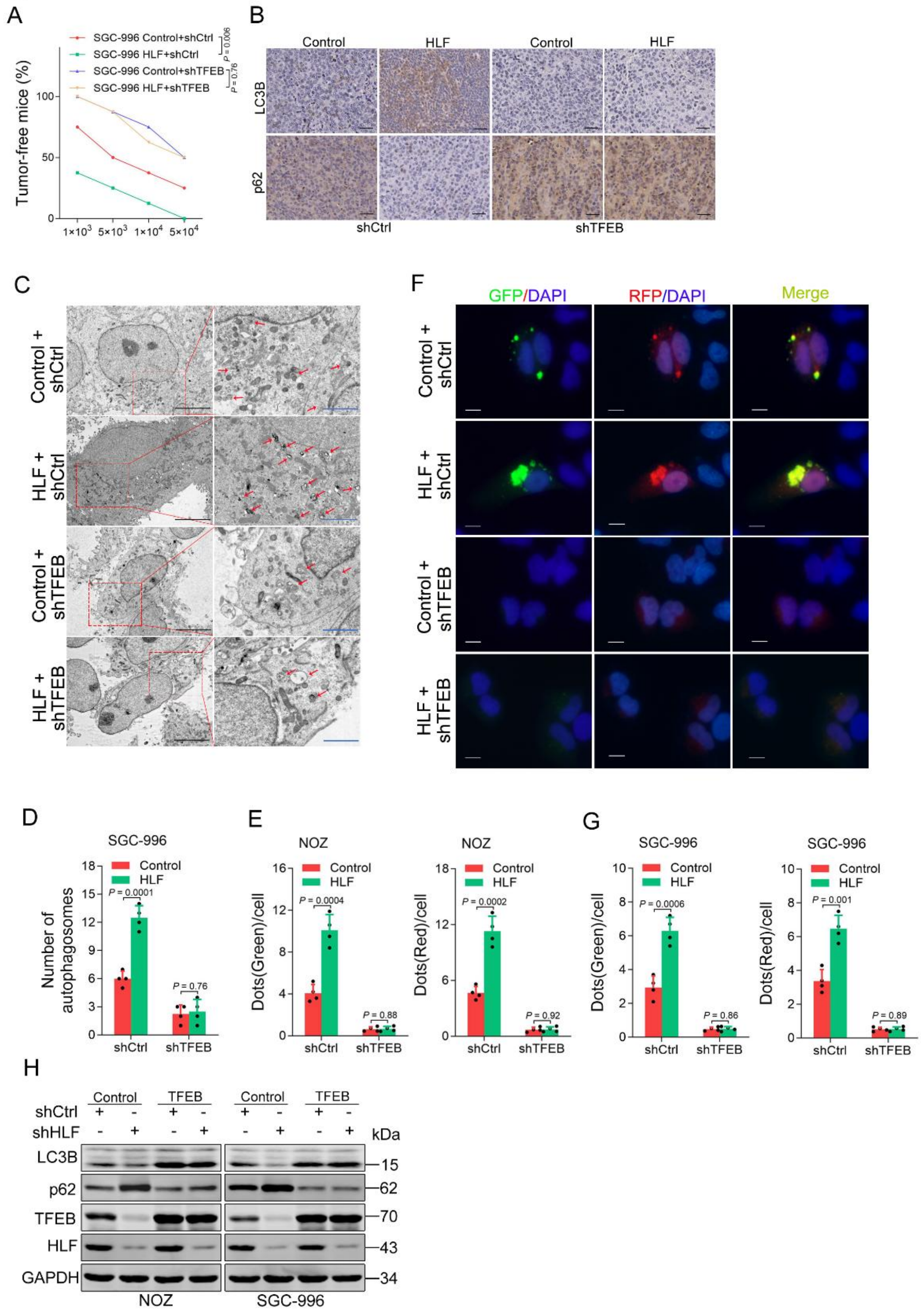

A
SGC-996 Control+shCtrl
SGC-996 HLF+shCtrl
SGC-996 Control+shTFEB
SGC-996 HLF+shTFEB
P = 0.006
P = 0.76
Tumor-free mice (%)
B
Control
HLF
LC3B
p62
shCtrl
shTFEB
C
Control + shCtrl
HLF + shCtrl
Control + shTFEB
HLF + shTFEB
F
GFP/DAPI
RFP/DAPI
Merge
D
SGC-996
Number of autophagosomes
P = 0.0001
P = 0.76
E
NOZ
Dots(Green)/cell
Dots(Red)/cell
P = 0.0004
P = 0.88
P = 0.0002
P = 0.92
G
SGC-996
P = 0.0006
P = 0.86
P = 0.001
P = 0.89
H
TFEB
shCtrl
shHLF
kDa
GAPDH
NOZ
SGC-996

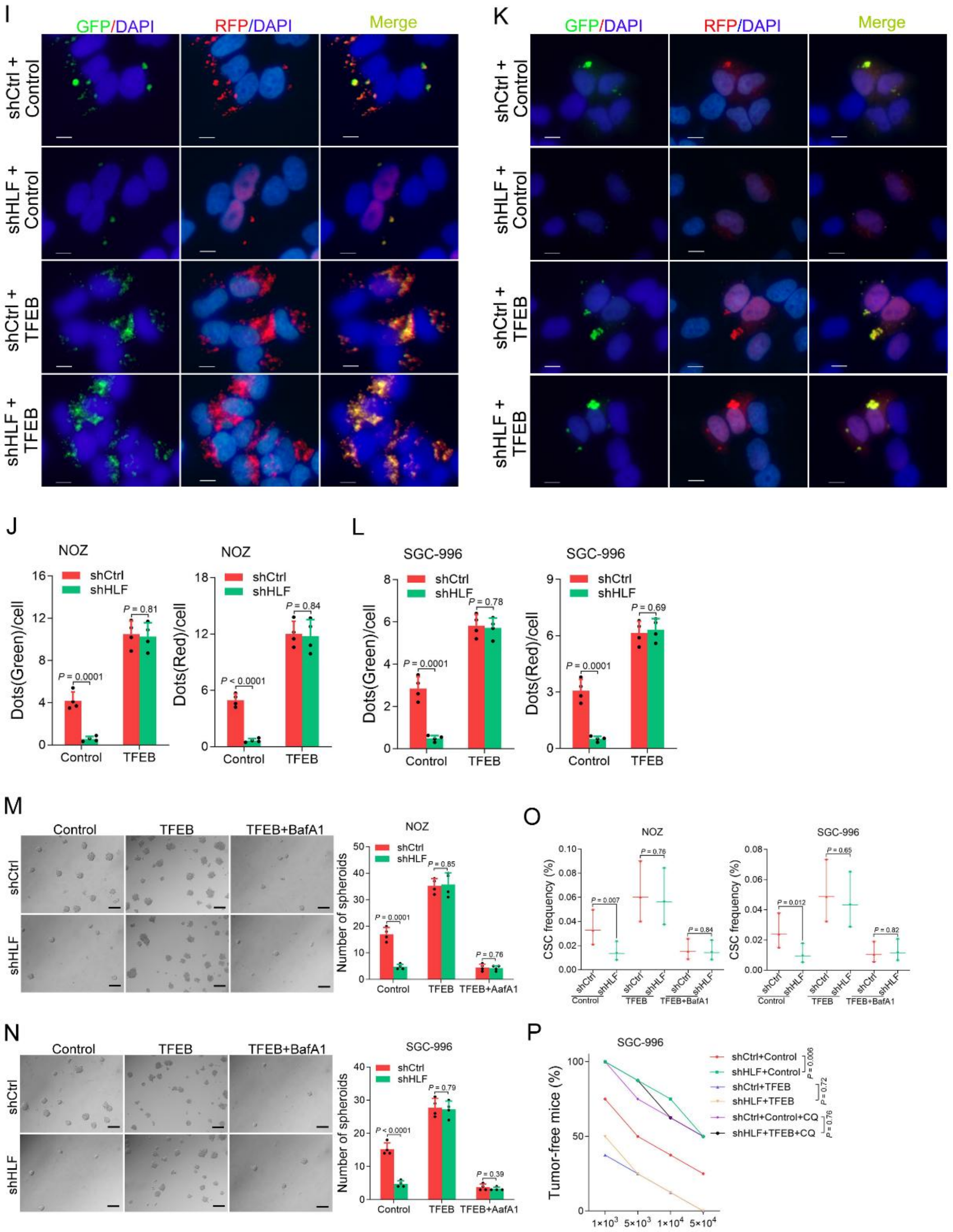

fig. S7 HLF promotes gallbladder CSCs self-renewal via TFEB.

(A) HLF overexpression and control cells infected with TFEB knockdown or control virus were subjected to *in vivo* limiting dilution assays.

(B) Immunohistochemical staining for LC3B and p62 in subcutaneous xenografts from (Fig. 4C). Scale bar, 25 μm.

(C and D) SGC-996 HLF and control cells infected with TFEB knockdown or control virus. Then the autophagosomes were observed under the transmission electron microscopy (TEM). Red arrows indicate the autophagosomes. Blue scale bar = 2 μm. Black scale bar = 5 μm.

(E) Statistical analysis of LC3 puncta in NOZ cells expressing mRFP-GFP-LC3.

(F and G) The effect of the HLF on promoting autophagy was blocked by shTFEB, as assessed by confocal imaging and the subsequent statistical analysis of LC3 puncta in SGC-996 cells expressing mRFP-GFP-LC3. Scale bar = 5 μm.

(H) NOZ/SGC-996 shHLF and control cells infected with TFEB overexpression or control virus were subjected to western blot assay.
(I-L) The effect of the shHLF on inhibiting autophagy was recovered by TFEB overexpressing, as assessed by confocal imaging and the subsequent statistical analysis of LC3 puncta in GBC cells expressing mRFP-GFP-LC3.
(M-O) shHLF and control cells were infected with *TFEB* overexpression virus. The indicated cells were subjected to spheroid formation (M and N) and *in vitro* limiting dilution assays (O) in presence and absence of BafA1 (10 nM) for 7 days. Bafilomycin A1 (BafA1) is an autophagy inhibitor.
(P) SGC-996 shHLF and control cells were infected with *TFEB* overexpression virus. The indicated cells were subjected to *in vivo* limiting dilution assays in presence and absence of CQ. 25 mg/kg CQ was injected intraperitoneally once daily for one week.

fig. S8

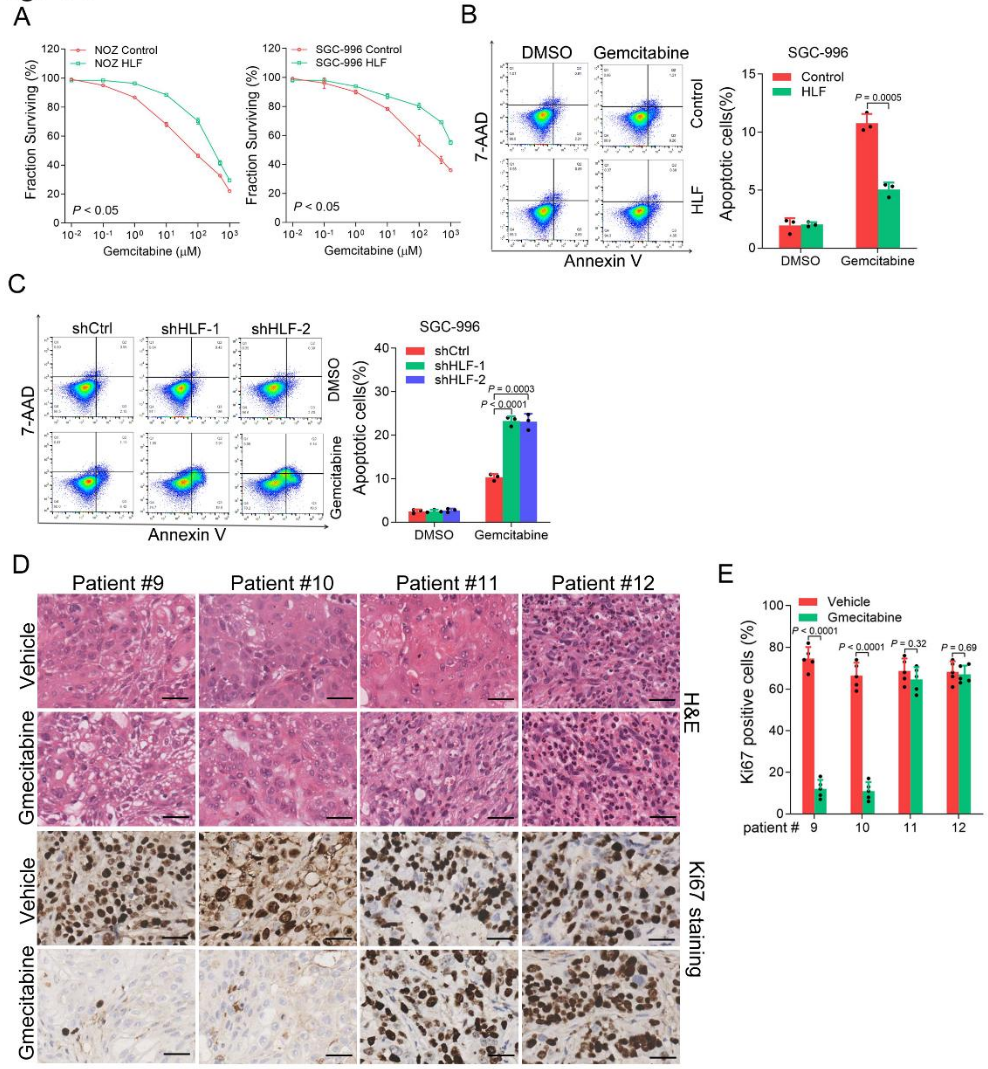

fig. S8 HLF facilitates gemcitabine resistance in GBC cells.

(A) HLF overexpression and control cells were treated with gemcitabine (0, 0.01, 0.1, 1, 10, 100, 500, 1000 μM) for 48 hours and cell survival curves were calculated.

(B) HLF overexpression and control SGC-996 cells were treated with gemcitabine for 48 hours, and apoptosis was examined by flow cytometry (n = 3).

(C) HLF knockdown and control GBC cells were treated with gemcitabine for 48 hours, and apoptosis was examined by flow cytometry (n = 3).

(D and E) PDXs derived from indicated patients treated with gemcitabine were subjected to Ki67 staining. Representative views were shown. The proportion of the Ki67 positive cells was quantified (n = 5). Scale bar = 25 μm.

fig. S9

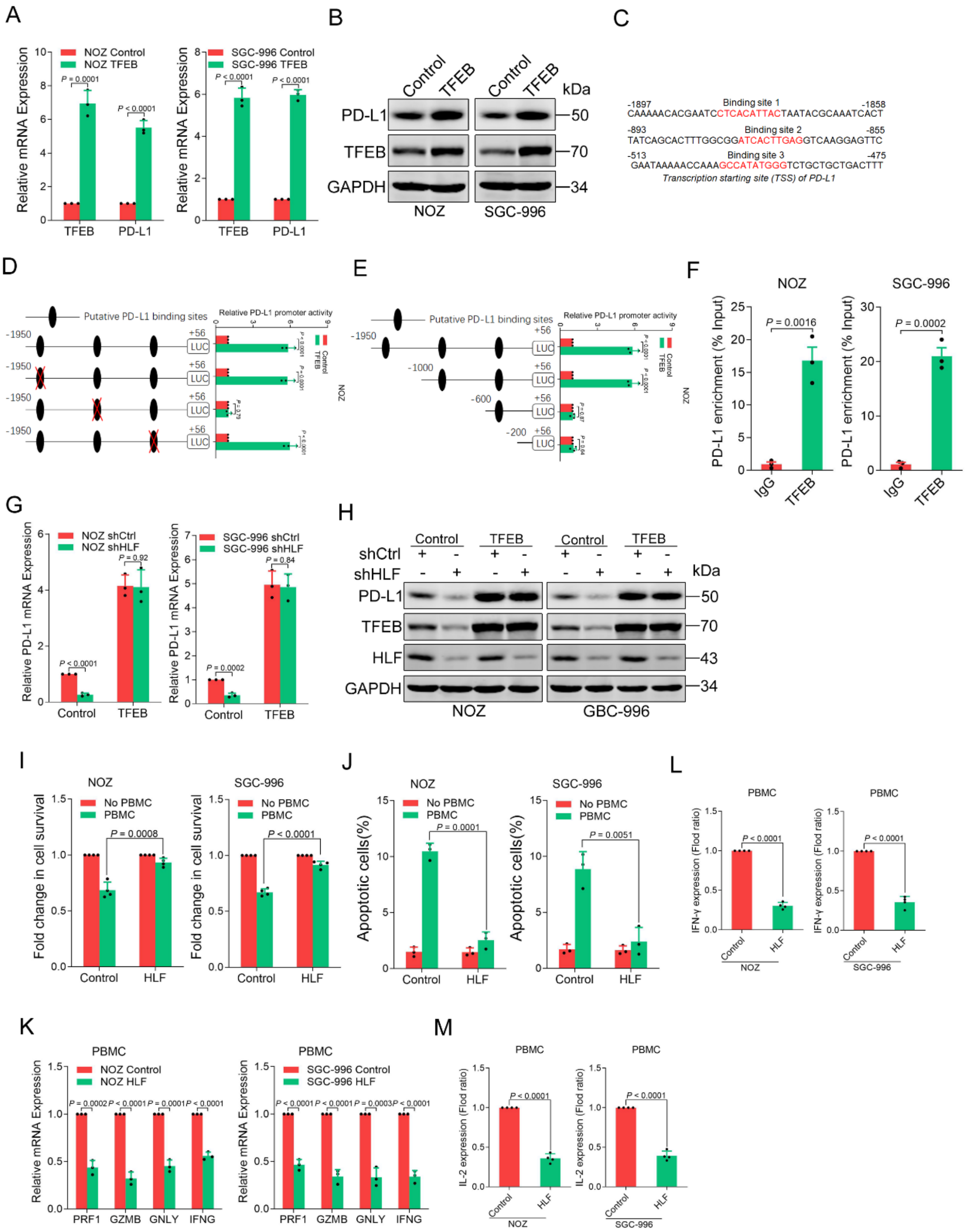

fig. S9 HLF/TFEB axis promotes GBC immune evasion.

(A) Real-time PCR analysis of the mRNA expression of PD-L1 in TFEB overexpression or control GBC cells (n = 3).

(B) Western blot analysis of the protein expression of PD-L1 in TFEB overexpression or control GBC cells.

(C) Potential binding sites of TFEB on PD-L1 promoter.

(D) Selective mutation analysis identified TFEB-responsive regions in the *PD-L1* promoter. Serially mutated PD-L1 promoter constructs were transfected into NOZ TFEB-overexpressing and control cells, and relative luciferase activities were determined (n = 3).

(E) Deletion analysis identified TFEB-responsive regions in the *PD-L1* promoter. Serially truncated

PD-L1 promoter constructs were transfected into NOZ TFEB overexpressing and control cells, and relative luciferase activities were determined (n = 3).

(F) GBC cells were subjected to ChIP assay with anti-TFEB or anti-IgG antibody followed by real-time PCR (n = 3).

(G) NOZ/SGC-996 shHLF and control cells infected with TFEB overexpressing or control virus were subjected to real-time PCR assay (n = 3).

(H) NOZ/SGC-996 shHLF and control cells infected with TFEB overexpressing or control virus were subjected to western blot assay.

(I) CCK8 assay detected the killing of tumor cells by activated PBMC. NOZ/SGC-996 HLF and control cells were co-cultured with or without PBMC for 48 h. Data were normalized to their respective no PBMC controls (n = 4).

(J) NOZ/SGC-996 HLF and control cells were co-cultured with or without PBMC for 48 h followed by cytometry analysis of apoptosis (n = 3).

(K) Real-time PCR was performed to detect PRF1, GZMB, GNLY and IFNG in activated PBMC co-cultured with NOZ/SGC-996 HLF and control cells (n = 3).

(L and M) Soluble INF-γ and IL-2 levels in the supernatants of co-cultures containing GBC cells and PBMCs as assayed using the ELISA assay (n = 4).

fig. S10

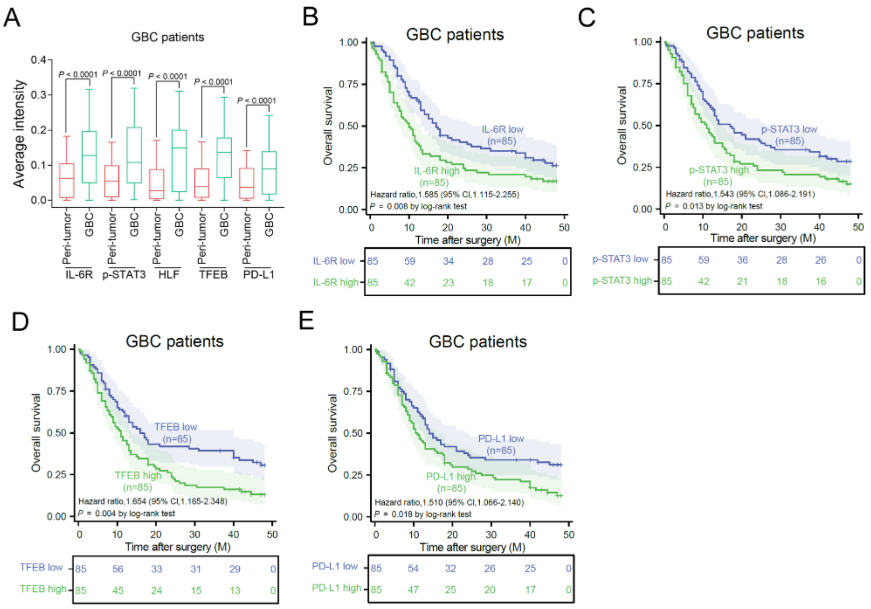

fig. S10 IL-6R/p-STAT3/HLF/TFEB/PD-L1 axis is activated in GBC and predicts poor prognosis.

(A) Immunohistochemical staining of IL-6R, p-STAT3, HLF, TFEB, and PD-L1 expression in tumor and the corresponding peri-tumor tissues from patients with GBC (n = 170). Quantification of indicated protein expression in tumor and the corresponding peri-tumor tissues from GBC patients. (B-E) Immunohistochemical staining analysis of IL-6R, p-STAT3, TFEB, and PDL-1 were performed in 170 GBC patients. Kaplan-Meier analysis for OS was performed according to IL-6R, p-STAT3, TFEB, and PDL-1 levels.

fig. S11

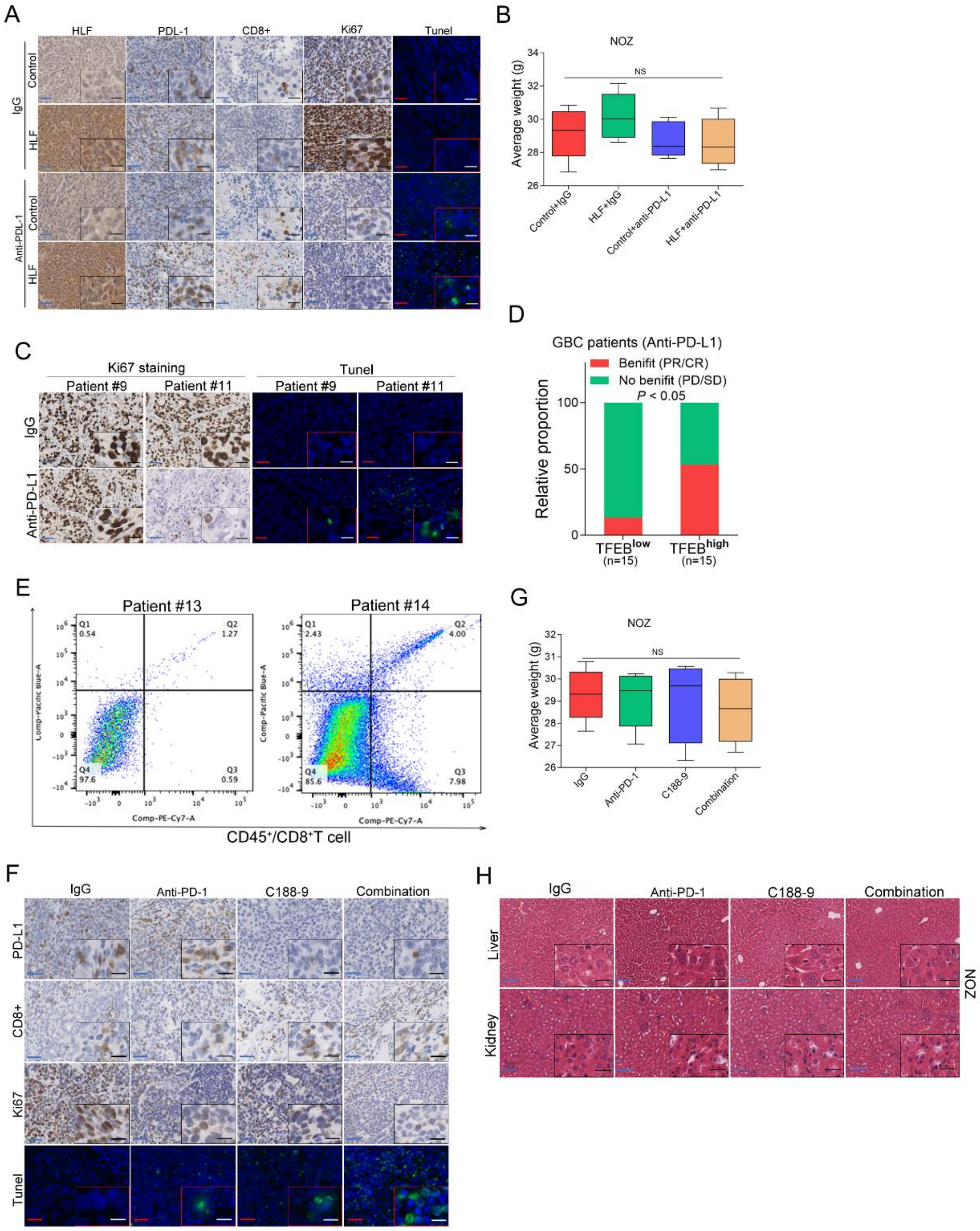

fig. S11 HLF expression associates the efficacy of PD-(L)1 checkpoint blockade in human tumors.

(A) Representative images of IHC staining of HLF, PD-L1, CD8, Ki67, and Tunel in xenografted tumors formed in (Fig. 7A). Blue scale bar = 5 μm. Black scale bar = 25 μm. Red scale bar = 5 μm. White scale bar = 25 μm.

(B) Average weight of mice (n = 5 per group) xenografts after indicated treatments. The horizontal lines in the box plots represent the medians, the boxes represent the interquartile range, and the whiskers represent the minimum and maximum values.

(C) Representative images of Ki67 and TUNEL staining in xenografted tumors formed PDXs (Fig. 7C).

(D) Correlation analysis of TFEB expression and responses of patients with GBC to anti-PD-1

treatment (n = 30).
(E) PDOs derived from the primary GBCs with low HLF levels (Patient #13) or high HLF levels (Patient #14) were co-cultured with activated PBMCs. Then the percent of $CD45^{+}/CD8^{+}$ T cells was examined by flow cytometry.
(F) Representative images of IHC staining of PD-L1, CD8, Ki67, and Tunel in xenografted tumors formed in (Fig. 7H). Blue scale bar = 5 μm. Black scale bar = 25 μm. Red scale bar = 5 μm. White scale bar = 25 μm.
(G) Average weight of mice (n = 5 per group) xenografts after indicated treatments. The horizontal lines in the box plots represent the medians, the boxes represent the interquartile range, and the whiskers represent the minimum and maximum values.
(H) H&E staining of mice organs was performed after indicated therapy. Blue scale bar = 5 μm. Black scale bar = 25 μm.

Supplementary tables

Table S1. Clinicopathologic Features of 170 GBC Specimens in Cohort 1

Characteristics	Cases	HLF expression		χ^2	*P*
		Low (n=85)	High (n=85)		
Age(year)				0.182	0.670
≤50	26	12	14		
>50	144	73	71		
Gender				5.689	**0.017**
Male	85	35	50		
Female	84	50	34		
Gallbladder stone				15.908	**＜0.001**
Absent	84	55	29		
Present	86	30	56		
Histology differentiation				21.502	**＜0.001**
Poor	61	16	45		
Well/moderate	109	69	40		
Lymph node metastasis				13.600	**＜0.001**
No	90	57	33		
Yes	80	28	52		
Liver metastasis				6.065	**0.014**
No	78	47	31		
Yes	92	38	54		
TNM				8.497	**0.004**
I-II	23	18	5		
III-IV	147	67	80		

TNM, Tumor-Nodes-Metastasis. “Liver metastasis” was defined as perihepatic liver metastasis.

Table S2. Clinicopathologic Features of 209 GBC Specimens in Cohort 2

Characteristics	Cases	HLF expression	χ^2	*P*

		Low (n=105)	High (n=104)		
Age(year)				0.067	0.796
<60	62	32	30		
⩾60	147	73	74		
Gender				1.137	0.286
Male	73	33	40		
Female	136	72	64		
Histology differentiation				6.655	0.010
Poor	124	50	74		
Well/moderate	95	55	40		
Lymph node metastasis				10.308	0.001
Yes	128	53	75		
No	81	52	29		
Nerve invasion				8.850	0.003
Yes	99	39	60		
No	110	66	44		
TNM				9.124	0.003
I-II	51	35	16		
III-IV	158	70	88		

TNM, Tumor-Nodes-Metastasis.

Table S3. Clinicopathologic features of 60 GBC specimens in cohort 3

Characteristics		n=60
Age(year)	⩽50	23
	>50	37
Gender	Male	21
	Female	39
Gallbladder stone	Present	40
	Absent	20
Histology differentiation	Poor	42
	Well/moderate	18
Lymph node metastasis	Yes	22
	No	38

Liver metastasis	Yes	45
	No	15
TNM	I-II	12
	III-IV	48

TNM, Tumor-Nodes-Metastasis.

Table S4. Clinicopathologic features of 30 GBC specimens in cohort 4

Characteristics		n=30
Age(year)	≤50	10
	>50	20
Gender	Male	11
	Female	19
Gallbladder stone	Present	22
	Absent	8
Histology differentiation	Poor	26
	Well/moderate	4
Lymph node metastasis	Yes	7
	No	23
Liver metastasis	Yes	15
	No	15
TNM	I-II	13
	III-IV	17

TNM, Tumor-Nodes-Metastasis.

Table S5. Antibody List

**Antigens**	**Manufacturer**	**Application**
HLF	Abcam (ab91630), USA	1:1000 for WB
HLF	The International Cooperation Laboratory on Signal Transduction, EHBH, SMMU, China	1:100 for IHC or 1:50 for IF
IL-6R	Proteintech Group (23457-1-AP), China	1:1000 for WB or 1:100 for IHC or 1:50 for IF
TFEB	Proteintech Group (13372-1-AP), China	1:1000 for WB or 1:100 for IHC or 1:50 for IF

PD-L1	Cell Signaling Technology (#13684), Beverly, MA	1:1000 for WB or 1:100 for IHC
P62	Proteintech Group (18420-1-AP), China	1:1000 for WB
LC3B	Cell Signaling Technology (#3868), Beverly, MA	1:1000 for WB
Ki67	Proteintech Group (27309-1-AP), China	1:100 for IHC
CD8	Cell Signaling Technology (#98941), Beverly, MA	1:100 for IHC
Flag	Abcam (ab205606), Cambridge, MA	1:1000 for WB or 1:50 for IP
p-STAT3	Abcam (ab76315), Cambridge, MA	1:1000 for WB or 1:100 for IHC
STAT3	Proteintech Group (60199-1-Ig), China	1:1000 for WB
p-JAK2	Cell Signaling Technology (#3771), Beverly, MA	1:1000 for WB
JAK2	Cell Signaling Technology (#3230), Beverly, MA	1:1000 for WB
GAPDH	Proteintech Group, China	1:5000 for WB

Table S6. Primer list

**Gene**	**Forward primer**	**Reverse primer (5'-3')**
HLF(Human)	Forward (5′- 3′)	ACCAAGTCCCATTGATCCTG
	Reverse (5′- 3′)	GCCCAGTACTTGTCATCCTTC
IL-6R(Human)	Forward (5′- 3′)	GGTCAAGGACCTCCAGCATC
	Reverse (5′- 3′)	CTGGATTCTGTCCAAGGCGT
TFEB(Human)	Forward (5′- 3′)	TCCAACAAGGGAAGGTGACAT
	Reverse (5′- 3′)	CAGCCTGAGCTTGCTGTCAT
PD-L1(Human)	Forward (5′- 3′)	GCAGGGCATTCCAGAAAGATG
	Reverse (5′- 3′)	TTGTATGGGGCGTTCAGCAA

PRF1(Human)	Forward (5′- 3′)	GGGATTCCAGAGCCCAAGTG
	Reverse (5′- 3′)	GAGAAGGATGCCCAGGAGGA
GZMB(Human)	Forward (5′- 3′)	GAGAAGGATGCCCAGGAGGA
	Reverse (5′- 3′)	AGATAAGCCATGTAGGGGCG
GNLY(Human)	Forward (5′- 3′)	AGGCTCCCTGCCCATAAAAC
	Reverse (5′- 3′)	CTCAAGGCCTGGGTTGCC
IFNG(Human)	Forward (5′- 3′)	ACTGTCGCCAGCAGCTAAAA
	Reverse (5′- 3′)	TATTGCAGGCAGGACAACCA
CD79B(Human)	Forward (5′- 3′)	AGAGCCCACGTTTCATAGCC
	Reverse (5′- 3′)	TCCAGAGCCAGCTCACATTG
THEGL(Human)	Forward (5′- 3′)	AATAAGAGGGCTCCTGTGCG
	Reverse (5′- 3′)	GGGTCTGACTGTTGTGGCTT
GAPDH (Human)	Forward (5′- 3′)	AATGGGCAGCCGTTAGGAAA
	Reverse (5′- 3′)	GCGCCCAATACGACCAAATC
TSKS(Human)	Forward (5′- 3′)	TGAACCTCAAACGGTCCTCG
	Reverse (5′- 3′)	TTTCCGTGATGTCTGCGTCA
DCLK1(Human)	Forward (5′- 3′)	TTGGCTTAGTGCTGAGACGG
	Forward (5′- 3′)	AGAGAGCTGACTACCAGGGG
RSPH4A(Human)	Forward (5′- 3′)	GGGGCTTCCTCTTTTGACAC
	Reverse (5′- 3′)	ATAACCGTGTTGTCCAGGGG
TMEM119(Human)	Forward (5′- 3′)	CAGCACGGACTCTCTCTTCC
	Reverse (5′- 3′)	GTGCCCCCAGGACCAGTTC
ANKH(Human)	Forward (5′- 3′)	GGCGGGGACTATGGTGAAAT
	Reverse (5′- 3′)	ACTGCATCCTCCTTGACAGC
AQP3(Human)	Forward (5′- 3′)	CTTCTTTGACCAGGACCGGC
	Reverse (5′- 3′)	GGGCCAGCTTCACATTCTCT
BAIAP2L2(Human)	Forward (5′- 3′)	CCTCCATCTCCAGGTCCCTC
	Reverse (5′- 3′)	TAGGTAGTCCCTCAGGTGCC
CYGB(Human)	Forward (5′- 3′)	GAAGACCCTCCTTGACGCTC
	Reverse (5′- 3′)	CCTCCTTCGGGGAAGTTGAG
NFIL3(Human)	Forward (5′- 3′)	GGCGCCGGGACATTTTAATC
	Reverse (5′- 3′)	AGAGGAGCCCTCTACCTTGG
TSPAN15(Human)	Forward (5′- 3′)	CCACCGTGTTCTGGACCATT
	Reverse (5′- 3′)	CACTGCAGTCGTGGTACTGA

ROGDI(Human)	Forward (5′- 3′)	CTGGGGCCATGTTCGAGTG
	Reverse (5′- 3′)	GTGAAGTAGACCAGGGCGTC
C1R(Human)	Forward (5′- 3′)	ACATGTCTGTGAGGTTCAGGG
	Reverse (5′- 3′)	GGCACCAGGAGGTACAAGAG
CYS1(Human)	Forward (5′- 3′)	GGGAGAGGGTCTGCCTGATA
	Reverse (5′- 3′)	ATTCCAAGGCTCTTCCTGCC
AHNAK(Human)	Forward (5′- 3′)	CTCAGGGAGGAGTGGTTGTG
	Reverse (5′- 3′)	TTTGCAGGATTCCGCTCAGG
BTN3A3(Human)	Forward (5′- 3′)	CAGAGTGCACCGTATCGAGG
	Reverse (5′- 3′)	TCCTCCACTCTGCAACCTTC
shHLF-1	5’- UGGGCAAAUGCAAGAACAUTT -3’	
shHLF-2	5’- GCTGGGCAAATGCAAGAACAT -3’	
shTFEB	5’- GGAUCAAGGAGCUGGGAAUUU -3’	
shIL-6R	5’-CCACGACUCUGGAAACUAUdTdT-3’	
